# Manipulation of regulators of morphogenesis is not sufficient to render a *Candida albicans* colonizer strain pathogenic

**DOI:** 10.1128/mbio.00415-26

**Published:** 2026-04-07

**Authors:** Ricardo Fróis-Martins, Sarah Mertens, Van Du T. Tran, Corinne Maufrais, Christophe d'Enfert, Dominique Sanglard, Salomé LeibundGut-Landmann

**Affiliations:** 1 Section of Immunology, Vetsuisse Faculty, University of Zurich 27217, Zurich, Switzerland; 2 Institute of Experimental Immunology, University of Zurich 27217, Zurich, Switzerland; 3 Vital-IT, SIB Swiss Institute of Bioinformatics 30489, Lausanne, Switzerland; 4 Institut Pasteur, Université Paris Cité, INRAe USC2019, Inserm U1359, Unité Biologie et Pathogénicité Fongiques 27058, Paris, France; 5 Institute of Microbiology, University of Lausanne and University Hospital Center 27213, Lausanne, Switzerland; 6 Medical Research Council Centre for Medical Mycology at the University of Exeter, Department of Biosciences, Faculty of Health and Life Sciences 3286, Exeter, United Kingdom; Instituto Carlos Chagas, Curitiba, Brazil

**Keywords:** *Candida albicans*, oral colonization, pathogenicity, inflammation hyphal network, cAMP-PKA pathway, *BRG1*

## Abstract

As a member of the microbiome, *Candida albicans* colonizes the oral cavity and other mucosal surfaces of the human body. While commensalism is tightly controlled by the host immune system, the fungal determinants enabling the fungus to colonize the host mucosa without causing tissue damage and inflammation remain less clear. In search of genetic determinants that may underlie the commensal lifestyle of the low-damage-inducing *C. albicans* isolate 101, we identified a small sequence duplication in one allele of the Brg1 hyphal morphogenesis regulator gene, resulting in a truncated loss-of-function allele (*BRG1*^TRUNC^). Replacing *BRG1*^TRUNC^ by the full-length allele (*BRG1^FL^*) resulted in a modest increase in filamentation but did not alter the phenotype of the fungus in the oral mucosa of experimentally colonized mice. Analysis of a spontaneous hyperfilamentous variant of the *BRG1^FL^*/*BRG1^FL^* derivative of *C. albicans* strain 101 identified a Glu-to-Lys change at position 1541 in the Cyr1 adenylate cyclase (*CYR1^E1541K^*). While the *CYR1^E1541K^* mutation led to greatly increased filamentation, expression of hyphae-associated genes, and host cell damage when tested *in vitro*, it was insufficient to render *C. albicans* strain 101 more pathogenic in the oral mucosa *in vivo*, irrespective of the *BRG1* status. Together, this highlights that the low-damage-inducing nature of strain 101 cannot be overcome by manipulating *BRG1* and *CYR1*, two genes with known roles in *C. albicans* virulence.

**IMPORTANCE:**

During homeostasis, the fungus *Candida albicans* establishes mutualistic interactions with its human host. It can, however, also adopt a pathogenic state and cause infections with diverse clinical manifestations that pose a significant challenge for diagnosis and therapy. Understanding the fungal determinants that underlie *C. albicans* colonization under steady-state conditions may thus provide new avenues for modulating the fungus-host interaction in candidiasis patients to restore homeostasis. Here, we report gene variants in key regulators of *C. albicans* morphogenesis and virulence that distinguish strains with distinct capacity to drive inflammation and cause disease. Gene-exchange mutants provided evidence for the impact of a *BRG1* loss-of-function allele and a *CYR1* gain-of-function mutation toward *in vitro* biomarkers of fungal pathogenicity. However, *in vivo* in an experimental model of *C. albicans* oral colonization, none of these gene variants individually or in combination was sufficient to change the pathogenic state of the fungus. These findings indicate that *C. albicans* mucosal colonization is regulated by a complex gene network rather than by single genetic determinants.

## INTRODUCTION

*Candida albicans* is a common member of the microbiome in the oral cavity, the gastrointestinal tract, and the female reproductive tract (1). Despite its primarily commensal lifestyle, *C. albicans* can become pathogenic and is therefore also referred to as a pathobiont. Under conditions of compromised host immunity or upon dysbiosis, it can cause superficial infections that manifest as oral and vaginal thrush (2, 3). In rare cases, such as in neutropenic individuals, *C. albicans* translocates across the epithelial barrier and disseminates via the bloodstream to cause systemic disease, which is associated with a high mortality rate due to liver, spleen, and kidney failure, and/or fungal meningitis (2). The pathogenic impact of *C. albicans* further extends to non-infectious diseases, such as inflammatory bowel disease and ethanol-induced liver disease (4–8). Treatment options are limited, and the rise in antifungal resistance jeopardizes the effectiveness of the few available antifungal drugs (9). A better understanding of the mechanisms governing fungal pathogenicity is needed to guide new approaches for disease prevention and therapy by targeting fungal pathogenicity determinants rather than aiming at eradicating the organisms with fungicidal drugs.

A hallmark of *C. albicans* pathogenicity is the fungus’ capacity to undergo morphological changes and switch between yeast and hyphal forms. While the yeast form was originally primarily associated with colonization and the hyphal morphotype with pathogenicity, this dichotomy has been challenged by the observation that both yeast-locked and hyperfilamentous mutants are avirulent in experimental infection models (10–12). Moreover, we and others have recently found that homeostatic colonization of the oro-gastrointestinal tract also depends on the capacity of *C. albicans* to filament, whereas yeast-locked mutants are bad colonizers in eubiotic hosts (10, 13). Therefore, dynamic morphological changes are critical for both fungal colonization and disease. Limiting the degree of filamentation and the processes associated with filamentation is essential for stable colonization during steady state and for maintaining tissue homeostasis (13, 14).

*C. albicans* filamentation is regulated by environmental cues that are integrated by diverse signal transduction pathways, including the Cek1 MAPK pathway, the Rim101 pathway, and the cAMP-dependent protein kinase A (PKA) pathway (15, 16). The adenylate cyclase Cyr1 plays a central role in translating environmental cues into PKA activation, in part by Ras-dependent mechanisms (17). Cyr1 converts ATP into the secondary messenger cAMP, which leads to PKA activation and, in turn, phosphorylation of the master regulator of the yeast-to-hyphae transition, Efg1 (18). Activated Efg1 not only drives the core filamentation response in *C. albicans* but also induces expression of numerous genes, including *HWP1*, *ALS3*, and *ECE1*. These hyphae-associated genes mediate fungal adhesion, tissue invasion, and host cell lysis, key processes in the interaction of the fungus with the host culminating in infection and tissue damage (19–21).

To limit the expression of these pathogenicity traits, *C. albicans* possesses hyphal repressors such as Nrg1, a DNA-binding protein repressing filamentous growth via the Tup1 pathway (22, 23). The expression of *NRG1* itself is regulated by Brg1, which influences the stability of *NRG1* transcripts, thus controlling hyphae formation via a feedback circuit (11).

*C. albicans* exhibits a large intraspecies diversity (24) with strain-specific differences at the genomic, genetic, and epigenetic levels translating into phenotypic variations (25, 26). As such, the *C. albicans* species comprises a large spectrum of isolates differing in their intrinsic capacity to filament, express hyphae-associated genes, and cause host cell damage, which, in turn, is further modulated by environmental and host factors. Most experimental studies have been conducted with strain SC5314, although this strain is not fully representative of the species due to its intrinsic highly pathogenic properties (25, 26). When orally administered to mice, it causes an acute inflammatory response (27). More recently, different groups, including our own, started to explore the intraspecies diversity of *C. albicans* and the consequences of strain variations on the behavior of the fungus at the host interface (25, 28, 29). Pinpointing the molecular basis underlying phenotypic differences between strains is challenging given the number of genetic differences that often separate natural isolates of *C. albicans* (30).

The oral isolate 101 is a low-damage-inducing strain, obtained from a healthy volunteer, that exhibits decreased filamentation and lower tissue invasiveness compared to strain SC5314 and is suitable for studying long-term fungal mucosal colonization (25). It persistently colonizes the oral mucosa of immunocompetent, non-antibiotic-treated mice without causing tissue damage and inflammation, reminiscent of *C. albicans* homeostatic colonization in humans. Yet, T cell and IL-17 deficiencies cause overgrowth of isolate 101, leading to symptoms reminiscent of oral thrush (25, 31, 32). The intrinsically low-damage-inducing nature of the strain is explained by the strong attenuation of key virulence genes, including those linked to filamentation, owing to high expression of the transcriptional repressor gene *NRG1* (14). Deletion or repression of *NRG1* rendered strain 101 more pathogenic by de-repressing the hyphal program (14). In search of genetic determinants underlying the elevated expression of *NRG1* in strain 101, we identified a truncated *BRG1* allele that was not present in other *C. albicans* isolates. We defined the role of the truncated allele in fungus-host interactions *in vitro* and *in vivo*. Furthermore, we found that strain 101 spontaneously acquires mutations in the RAS/cAMP-PKA pathway, which de-repressed several pathogenicity traits. The strong induction of filamentation and *ECE1* expression observed under *in vitro* conditions was, however, not paralleled by increased pathogenicity *in vivo*. Instead, strain 101 variants preserved a largely colonizer behavior in the murine oral mucosa, highlighting that the low-damage-inducing nature of strain 101 cannot be overcome by manipulating individual genes.

## RESULTS

### The truncated *BRG1* allele identified in strain 101 is unable to drive pathogenicity of strain SC5314

In search of mutations that may underlie the colonizer phenotype of strain 101, we established and annotated the full genome of strain 101 (BioProject PRJNA923600). The 101 genome was resolved into two separate haplotypes (BioProjects PRJNA1074522 and PRJNA1074523; see Material and Methods for details) with each eight large contigs corresponding to the eight chromosomes of the reference SC5314. The two haplotypes contained 5,829 and 5,816 annotated genes, which is lower than SC5314 (6,213 annotated genes), primarily due to the absence of annotations for ORFs smaller than 500 bp in the 101 haplotypes. The two 101 haplotypes exhibit low heterozygosity (approximately 11,000 SNPs between each haplotype).

**Fig 1:**
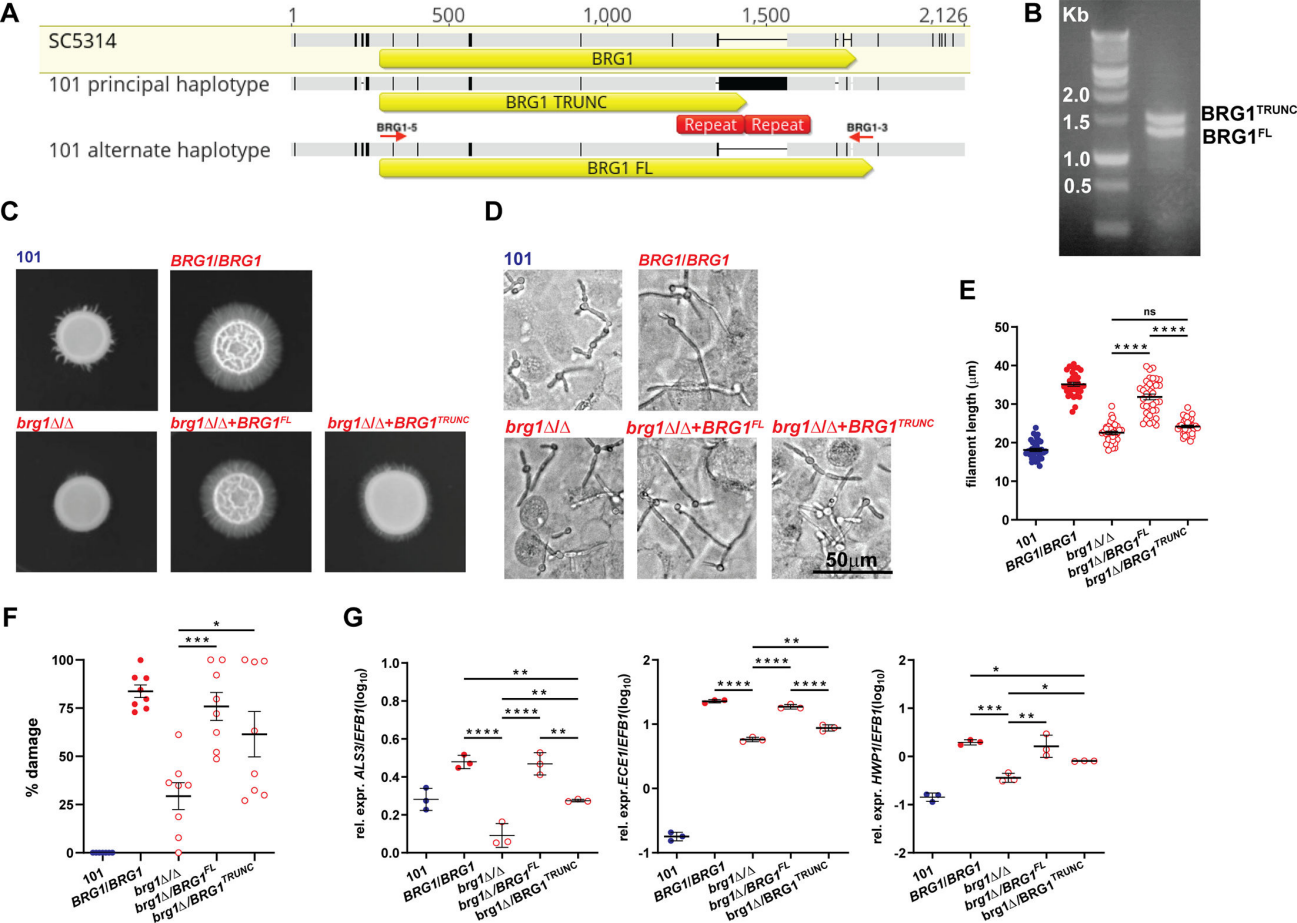
The truncated *BRG1* allele identified in strain 101 is unable to drive pathogenicity of strain SC5314. (**A and B**) Two different *BRG1* alleles are present in strain 101. (**A**) Alignment of the *BRG1* region of the two haplotypes of strain 101 compared to the *BRG1* locus (C1_05140W_A) of SC5314 as reference (yellow highlighted). *BRG1* open reading frames (ORF) are indicated by yellow arrows below each aligned nucleotide sequence. The polymorphisms between the different *BRG1* alleles are indicated by black bars in the grayed nucleotide sequences. The repeated regions (indicated by red rectangles) result in a 200 bp increase of PCR product for the *BGR1*^TRUNC^ allele (see panel **B**). Alignment was obtained with Geneious Prime (Biomatters, Ltd.). (**B**) PCR amplification of *BRG1* alleles in 101 and visualization of two different band sizes (1.5 and 1.3 kb). Primers BRG1-5 and BRG1-3 that were used in PCR are shown in panel **A** and are located at the boundaries of the *BRG1* ORF, resulting in 1.5 and 1.3 kb products for *BRG1*^TRUNC^ and *BRG1^FL^*, respectively, which is consistent with gel electrophoresis. Left lane: molecular weight standard (Benchtop 1 kb DNA Ladder, Promega). (**C–G**) *C. albicans* strains SC5314_*BRG1*/*BRG1*, SC5314_*brg1*Δ/Δ, SC5314_*brg1*∆/*BRG1*^FL^, SC5314_*brg1*∆/*BRG1*^TRUNC^ (red labels), and 101 (blue labels) were assessed for their phenotype *in vitro*. (**C**) Colony morphology of the strains grown on Spider agar for 7 days at 35°C. (**D**) Representative images of filamenting strains upon contact with a monolayer of TR146 keratinocytes for 3.5 h at 37°C, 5% CO_2_. (**E**) Quantification of the filament length. Each symbol represents the mean filament length of 20–30 filaments per field. Data are pooled from two independent experiments with 15–20 visual fields analyzed per strain each. The mean ± SEM is indicated. (**F**) LDH released from TR146 keratinocytes after exposure to the fungal strains for 24 h at 37°C, 5% CO_2_. Each symbol represents one well. Data are pooled from two independent experiments with four wells per strain each. The mean ± SEM is indicated. (**G**) *ECE1*, *HWP1*, and *ALS3* expression levels by the fungal strains after exposure to TR146 keratinocytes for 24 h at 37°C, 5% CO_2_. Each symbol represents one sample. Data are from one representative out of two independent experiments with three samples per strain each. The mean ± SD is indicated. Statistical significance was determined using one-way ANOVA. *P < 0.05, **P < 0.01, ***P < 0.001, ****P < 0.0001. See also Fig. S1.

While focusing on known *C. albicans* genes affecting filamentation and/or pathogenicity traits, we identified, using haplotype alignment, a 209-base-pair duplication at the 3′ end of the *BRG1* gene in one of the haplotypes, creating a truncated protein due to a frameshift (Fig. 1A and Fig. S1). Amplification by PCR of the *BRG1* alleles in strain 101 confirmed the presence of two distinct alleles that differ in length (Fig. 1B). The gene duplication responsible for the truncated *BRG1* allele in strain 101 (*BRG1*^TRUNC^) is unique across >250 whole-genome (short-read) sequenced isolates.

The second haplotype retained a full-length copy of the *BRG1* gene in strain 101 (termed *BRG1^FL^* in the following), which slightly differs from the two revised *BRG1* alleles of strain SC5314 (Fig. S1). Notably, our phasing of single nucleotide polymorphisms and analysis of insertion-deletion data at the *BRG1* locus for 182 *C. albicans* genome-sequenced isolates (24) revealed a frameshift in the *BRG1* haplotypes of strain SC5314 (Assembly 22). Correction of this frameshift and glutamine-rich coding regions led to two Brg1 proteins with C-terminal sequences identical to that in the Brg1-FL protein of strain 101 (Fig. S1). The same was observed for strains CEC3672 and CEC3678 that will be described below. Given the role of Brg1 as a transcription factor associated with *C. albicans* virulence and biofilm formation, acting as a negative regulator of Nrg1 (11, 33), and because of the causal link between the degree of *NRG1* expression and the colonizer phenotype of strain 101 (14), we decided to experimentally investigate the functional role of the newly identified truncated *BRG1* allele in *C. albicans* pathogenicity.

We started by introducing the truncated allele, or the full-length allele from strain 101 (*BRG1*^FL^) as a control, into a *BRG1* null mutant originating from the Homann deletion set (34)(SC5314_*brg1*Δ/Δ). *BRG1* expression levels were restored irrespective of the allele (Fig. S2A). While full deletion of *BRG1* strongly impacted filamentation, the introduction of one copy of the full-length *BRG1* allele of strain 101 into SC5314_*brg1*Δ*I*Δ was sufficient to restore filamentation on Spider agar (Fig. 1C), indicating that *BRG1*^FL^ of strain 101 is fully functional. In contrast, the truncated *BRG1* allele of strain 101 was unable to do so (Fig. 1C). Likewise, the filamentation defect of strain SC5314_*brg1*Δ*I*Δ when placed in contact with oral keratinocytes (TR146 cells) to mimic *in vitro* the host interface of the oral cavity (*in vitro* filamentation) was restored upon introduction of *BRG1*^FL^ in this strain, whereas it was not restored upon introduction of *BRG1*^TRUNC^ (Fig. 1D and E). Introduction of the *BRG1*^TRUNC^ allele into wild-type SC5314, replacing one full-length allele while leaving intact the second full-length allele, did not compromise filamentation under the same assay conditions, indicating that the *BRG1*^TRUNC^ allele did not act as a dominant-negative allele (Fig. S2B).

In line with the requirement of strong filamentation for epithelial cell damage induction by *C. albicans* SC5314 (19), introducing the *BRG1*^TRUNC^ allele into SC5314_*brg1*Δ/Δ only partially restored the high degree of damage in TR146 keratinocytes (*in vitro* damage induction) that was elicited in response to strains containing one or two copies of the *BRG1*^FL^ allele (Fig. 1F). Again, in a mutant strain containing both a *BRG1*^TRUNC^ and a *BRG1*^FL^ allele, the truncated allele did not alter the effect of the full-length allele (Fig. S2C).

These observations suggested that the manifestation of pathogenicity traits depended on *BRG1* integrity. This dependence extended to the expression of hyphae-associated genes by *C. albicans* in contact with TR146 keratinocytes (*in vitro* hyphae-associated gene expression), including the candidalysin-encoding *ECE1* gene and the adhesin-coding genes *ALS3* and *HWP1*, all of which are among the genes that were defined as constituting the core filamentation response (35). While the introduction of *BRG1*^FL^ into SC5314_*brg1*Δ/Δ restored gene expression levels similar to those in SC5314, the *BRG1*^TRUNC^ allele led to only limited expression of hyphae-associated genes (Fig. 1G). In the presence of a full-length gene copy, introduction of *BRG1*^TRUNC^ had no impact on the expression of hyphae-associated genes, again confirming that the truncated allele was not dominant-negative (Fig. S2D). Together, these results demonstrate that the truncated *BRG1* allele identified in strain 101 was dysfunctional in *C. albicans*. Although our experiments demonstrate that it does not act as a dominant-negative allele in SC5314, we speculate that in a low-damage-inducing strain like 101, it may impact the strain’s phenotype, even in the presence of an intact full-length allele.

### Restoring the full-length *BRG1* allele in strain 101 is insufficient to increase the strain’s pathogenicity

**Fig 2:**
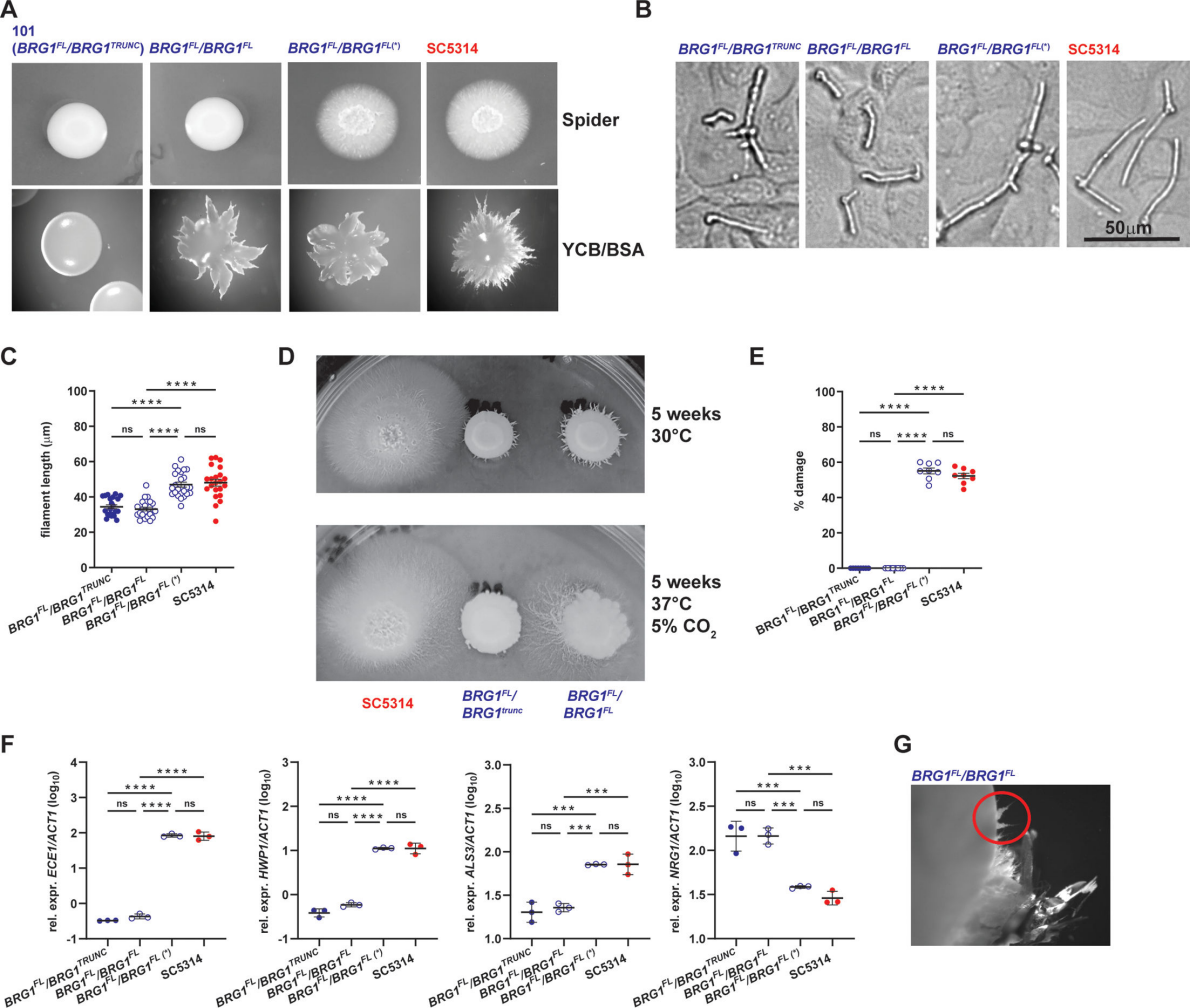
Restoring the full-length *BRG1* allele in strain 101 is insufficient to increase the strain’s pathogenicity when tested *in vitro*. *C. albicans* strains 101_*BRG1*^FL^/*BRG1*^TRUNC^, 101_*BRG1*^FL^/*BRG1*^FL^, 101_*BRG1*^FL^/*BRG1*^FL^(*), and SC5314 wild type were assessed for their phenotype *in vitro*. (**A**) Colony morphology of the strains grown on Spider agar (top) or on YCB/BSA agar (bottom) for 7 days at 35°C. (**B**) Representative images of filamenting strains upon contact with a monolayer of TR146 keratinocytes for 3.5 h at 37°C, 5% CO_2_. (**C**) Quantification of the filament length. Each symbol represents the mean filament length of 20–30 filaments per visual field. Data are pooled from two independent experiments with 10–12 visual fields analyzed per strain each. The mean ± SEM is indicated. (**D**) Colony morphology of the indicated strains grown on Spider agar for 5 weeks at 37°C, 5% CO_2_. (**E**) LDH release from TR146 keratinocytes after exposure to the fungal strains for 24 h at 37°C, 5% CO_2_. Each symbol represents one well. Data are pooled from two independent experiments with four wells per strain each. The mean ± SEM is indicated. (**F**) *ECE1*, *HWP1*, *ALS3*, and *NRG1* expression levels by the fungal strains after exposure to TR146 keratinocytes for 24 h at 37°C, 5% CO_2_. Each symbol represents one sample. Data are from one representative out of two independent experiments with three samples per strain each. The mean ± SD is indicated. (**G**) Close-up of a colony of 101_*BRG1*^FL^/*BRG1*^FL^ on Spider agar showing signs of filamentation in a subcolony. In panels **C, D, F, and G**, statistical significance was determined using one-way ANOVA. *P < 0.05, **P < 0.01, ***P < 0.001, ****P < 0.0001; ns, non-significant (*P* > 0.05). See also Fig. S2.

After finding that the *BRG1*^TRUNC^ allele of strain 101 is hypomorphic, while the full-length allele is fully functional in the context of SC5314 (Fig. 1), we speculated that replacing *BRG1*^TRUNC^ in strain 101 with a second copy of *BRG1*^FL^ may render this strain more pathogenic. Notably, a strain 101 derivative bearing two *BRG1*^FL^ alleles did not show any change in colony morphology on Spider agar compared to the parental strain 101, which bears *BRG1*^TRUNC^ and *BRG1*^FL^ alleles (Fig. 2A, top and Fig. S3). Likewise, the replacement of *BRG1*^TRUNC^ by *BRG1*^FL^ did not increase the degree of *in vitro* filamentation of strain 101 in contact with TR146 keratinocytes (Fig. 2B and C). Growth on agar, which also triggers filamentation (embedded growth), indicated that the allele replacement did impact fungal morphogenesis, although only under selected conditions (Fig. 2A**,** bottom). Moreover, longer incubation on Spider agar at 37°C revealed a mildly enhanced filamenting capacity of the mutant (Fig. 2D). However, the effect was not paralleled by a restoration of other pathogenicity traits. *In vitro* damage induction, which serves as a good correlate for *C. albicans* pathogenicity in barrier tissues (25), was not enhanced by replacing *BRG1*^TRUNC^ with *BRG1*^FL^ in strain 101 (Fig. 2E). Likewise, *in vitro* expression of the hyphae-associated genes *ECE1*, *HWP1*, and *ALS3* was not enhanced, and *NRG1* remained highly expressed (Fig. 2F). Together, these findings indicate that replacing the hypomorphic *BRG1*^TRUNC^ allele in strain 101 with a fully functional *BRG1* copy was not sufficient to alter the strain’s pathogenicity in terms of *in vitro* damage induction and hyphae-associated gene expression and only exerted a very limited effect in morphology.

### Spontaneous mutants convert strain 101 into a highly pathogenic variant

While performing *in vitro* experiments with strains 101_*BRG1*^FL^/*BRG1*^TRUNC^ and 101_*BRG1*^FL^/*BRG1*^FL^ shown in Fig. 2A through F, we observed that the latter strain, when grown on Spider agar, displayed some spikes at the border of the colony, resembling hyphal outgrowth (Fig. 2G). Material from the spike was collected and diluted to obtain single colonies. A clone generated from these spontaneously outgrown colonies, termed 101_*BRG1*^FL^/*BRG1*^FL^(*), displayed a highly filamentous morphology under all conditions tested. On Spider agar, in suspension culture, and when put in contact with keratinocytes, the strain closely resembled strain SC5314 (Fig. 2A through C and Fig. S3). It also elicited strong *in vitro* damage in TR146 cells (Fig. 2E), and it displayed an *in vitro* gene expression profile strikingly similar to that of strain SC5314 (Fig. 2F). Together, these findings indicate that a low-damage-inducing strain can convert into a high-damage-inducing variant, as inferred from *in vitro* conditions.

To further investigate the pathogenicity of the spontaneously outgrown 101_*BRG1*^FL^/*BRG1*^FL^(*) strain at the interface with the host, we made use of our experimental model of oral colonization in mice. In this model, *C. albicans* strain 101 efficiently establishes colonization of the epithelium, as assessed by quantification of the fungal burden in the tongue and by histological analysis of the tongue epithelium (25). In contrast, high-damage-inducing strains such as SC5314 swiftly induce a strong inflammatory response upon invasion of fungal hyphae into the nucleated layers of the epithelium (stratum granulosum, stratum spinosum), where they stimulate cytokine and chemokine production, which in turn results in an accumulation of inflammatory cells. This massive response, which peaks between day 1 and day 2 post-infection, leads to clearance of the fungus within 3–5 days (27, 36, 37). In contrast, low-damage-inducing strains such as 101 reside predominantly in the stratum corneum and elicit a non-inflammatory, homeostatic response characterized by tissue-resident Th17 cells (13, 31). *C. albicans*-specific Th17 cells mediate immunosurveillance to prevent fungal overgrowth, without eliminating the fungus, thereby allowing long-lasting colonization, which mimics *C. albicans* colonization in humans. This model is therefore ideally suited for assessing and distinguishing high- and low-damage-inducing isolates of *C. albicans* (25).

**Fig 3:**
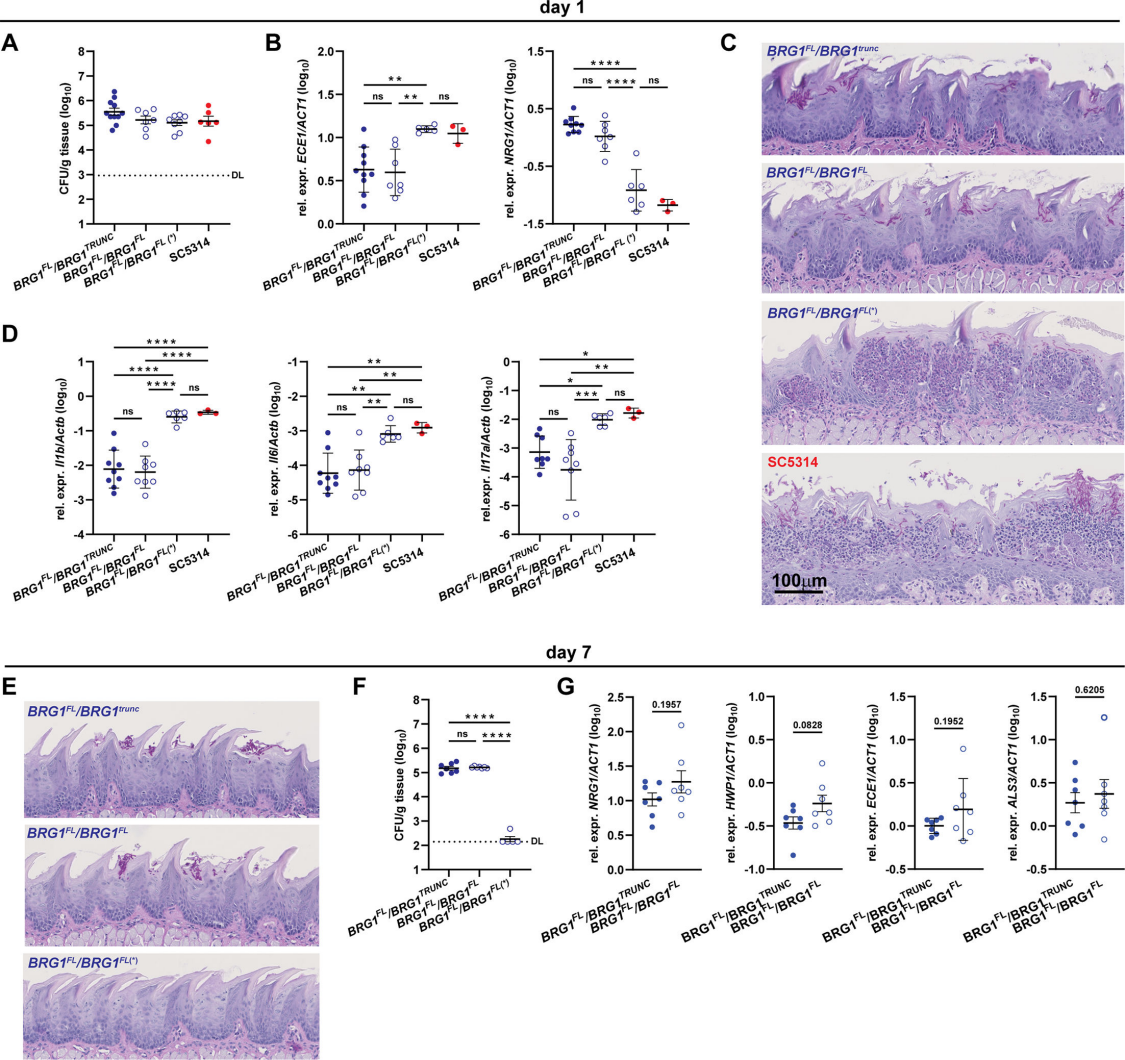
Restoring the full-length *BRG1* allele in strain 101 is insufficient to increase the strain’s pathogenicity *in vivo*. C57BL/6 mice were colonized with *C. albicans* strains 101_*BRG1*^FL^/*BRG1*^TRUNC^, 101_*BRG1*^FL^/*BRG1*^FL^, 101_*BRG1*^FL^/*BRG1*^FL^(*), and SC5314 wild type for 1 day (**A–D**) or 7 days (**E–G**). (**A**) Tongue fungal burden on day 1 after colonization. DL, detection limit. (**B**) *ECE1* and *NRG1* expression levels in the tongue tissue on day 1 after colonization. (**C**) PAS-stained histology sections of the tongue on day 1 after colonization. (**D**) Cytokine expression levels in the tongue tissue on day 1 after colonization. (**E**) PAS-stained histology sections of the tongue on day 7 after colonization. (**F**) Tongue fungal burden on day 7 after colonization. DL, detection limit. (**G**) *ECE1*, *ALS3*, *HWP1,* and *NRG1* expression levels in the tongue tissue on day 7 after colonization. In** panels A, B, D, E**, and **G**, each symbol represents one animal. Data are pooled from at least two independent experiments except for SC5314 in panels **B** and **D**. The mean ± SEM is indicated. Statistical significance was determined using two-way ANOVA (**B, D, E**) or unpaired Student’s *t*-test (**G**). *P < 0.05, **P < 0.01, ***P < 0.001, ****P < 0.0001. See also Fig. S3.

We colonized wild-type C57BL/6 mice with strain 101 carrying one or two *BRG1*^FL^ alleles, as well as strain 101_*BRG1*^FL^/*BRG1*^FL^(*), in comparison to SC5314. All strains colonized the tongue with comparable efficiency (Fig. 3A). Reminiscent of what we observed *in vitro*, *in vivo ECE1* expression was strongly induced and *NRG1* expression repressed in 101_*BRG1*^FL^/*BRG1*^FL^(*), but not in 101_*BRG1*^FL^/*BRG1*^TRUNC^ or 101_*BRG1*^FL^/*BRG1*^FL^ (Fig. 3B). Strong induction of hyphae-associated gene expression correlated with the activation of a pronounced inflammatory response. Large agglomerates of inflammatory cells accumulated in the tongue epithelium of mice colonized with high-damage-inducing isolates within as little as 1 day after infection (Fig. 3C). Concomitantly, we observed expression of inflammatory cytokines in the colonized tissue (Fig. 3D). Again, this response was limited to the spontaneously arisen, high-damage-inducing mutant, and its extent was comparable to that of the well-characterized strain SC5314. Reminiscent of what is known for SC5314, strain 101_*BRG1*^FL^/*BRG1*^FL^(*) did not persist, but was rapidly cleared from the tongue epithelium, despite its origin from the persistent colonizer 101 (Fig. 3E). Together, this indicates that spontaneous changes converted a colonizer strain into a highly pathogenic one that phenocopies SC5314 in all parameters tested.

The mutant of 101 carrying two full-length *BRG1* alleles could not be distinguished from its parental strain, which carries only one fully functional *BRG1* allele, in terms of its behavior in the murine tongue tissue based on fungal burden, histology, and *in vivo* expression of hyphae-associated fungal genes and host genes on day 1 post-infection (Fig. 3A through D). Accordingly, the strain persisted in the tongue, as did the parental 101_*BRG1*^FL^/*BRG1*^TRUNC^, with high fungal burden one week after infection (Fig. 3E and F). Of note, no significant differences were detected in the expression of *ECE1*, *HWP1*, and *ALS3* in the tongue colonized with strain 101_*BRG1*^FL^/*BRG1*^FL^ in comparison to the parental 101_*BRG1*^FL^/*BRG1*^TRUNC^ on day 7 post-infection (Fig. 3G).

### *CYR1*^E1541K^ induces the expression of virulence traits in strain 101

We hypothesized that the highly pathogenic phenotype of strain 101_*BRG1*^FL^/*BRG1*^FL^(*) was the result of spontaneous mutations arising during growth of strain 101_*BRG1*^FL^/*BRG1*^FL^ on Spider medium. To identify such mutation(s), whole-genome sequencing of strains 101_*BRG1*^FL^/*BRG1*^FL^ and 101_*BRG1*^FL^/*BRG1*^FL^(*) was performed, and the short reads for both strains were aligned to the reference genome. Omitting polymorphisms that had already been identified in a set of 298 clinical isolates, including high- and low-damage-inducing strains, and focusing on SNPs resulting in a non-synonymous amino-acid change, we identified 17 SNPs that distinguished the two strains (Table S1). Fourteen SNPs were found in cell wall protein-coding genes (*ALS2*, *ALS4*, *PGA23*, *FGR23/PGA40*, *C1_05970W*, *C3_01260C*), as well as in the *FGR28* and *NAG3* genes, all of which are prone to sequencing errors because of repeated regions. One SNP was found in the *CR_00470W* gene of unknown function. Finally, we noticed a non-synonymous SNP in the gene encoding the adenylyl cyclase Cyr1, which leads to a Glu to Lys substitution in a conserved C-terminal motif close to the protein’s catalytic domain (position 1541). As an integral part of the cAMP-PKA pathway, Cyr1 regulates *C. albicans* morphogenesis (18). In response to environmental cues, it generates short-lived intracellular cAMP spikes. The Glu to Lys substitution at position 1541 of Cyr1 has been identified previously in a genetic screen for SC5314 derivatives with increased Cyr1 activity. This mutation renders the enzyme hyperactive and causes constitutive filamentous growth of *C. albicans* SC5314 even under non-hyphae-inducing conditions (38).

**Fig 4:**
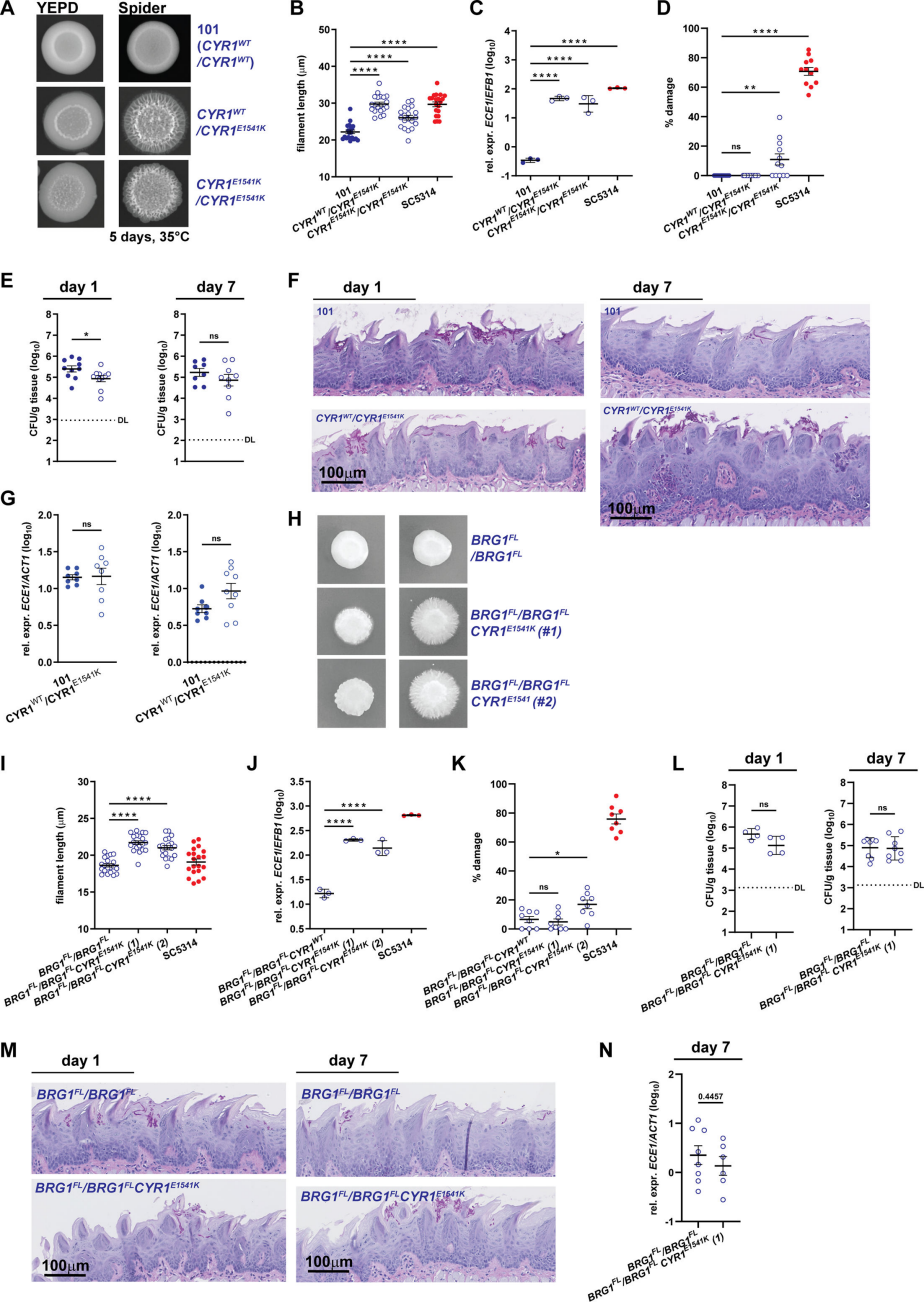
*CYR1*^E1541K^ induces the expression of pathogenicity traits in strain 101. (**A–D**). *C. albicans* strains 101 wild type, 101_*CYR1*^E1541K^, and 101_*CYR1*^E1541K/E1541K^ were assessed for their phenotype *in vitro*. (**A**) Colony morphology of the strains grown on Spider agar for 5 days at 35°C. (**B**) Filamentation of the strains upon contact with a monolayer of TR146 keratinocytes for 3.5 h at 37°C, 5% CO_2_. Each symbol represents the mean filament length of 20-30 filaments per visual field. The mean ± SEM is indicated. (**C**) *ECE1* expression levels by the fungal strains after exposure to TR146 keratinocytes for 24 h at 37°C, 5% CO_2_. Each symbol represents one sample. Data are from one representative out of two independent experiments with three samples per strain each. The mean ± SD is indicated. (**D**) LDH release from TR146 keratinocytes after exposure to the fungal strains for 24 h at 37°C, 5% CO_2_. Each symbol represents one well. Data are pooled from two independent experiments. The mean ± SEM is indicated. (**E-G**) C57BL/6 mice were colonized with *C. albicans* strains 101 wild type or 101_*CYR1*^E1541K^ and tongue fungal burden (**E**), PAS-stained histology sections of the tongue (**F**), and *ECE1* expression levels in the tongue tissue (**G**) were analyzed on day 1 and day 7 after colonization. Each symbol represents one mouse. Data are pooled from two independent experiments. The mean ± SEM is indicated. DL, detection limit. (**H–K**) *C. albicans* strains 101_*BRG1*^FL/^*BRG1*^FL^, 101_*BRG1*^FL/^*BRG1*^FL^ *CYR1*^E1541K^ (clone #1 and clone #2), and SC5314 wild type were assessed for their phenotype *in vitro*. (**H**) Colony morphology of the strains grown on Spider agar for 5 days at 37°C**.** (**I**) Filamentation of the strains put in contact with a monolayer of TR146 keratinocytes for 3.5 h at 37°C, 5% CO_2_. Each symbol represents the mean filament length of 20–-filaments per visual field. The mean ± SEM is indicated. (**J**) *ECE1* expression levels by the fungal strains after exposure to TR146 keratinocytes for 24 h at 37°C, 5% CO_2_. Each symbol represents one sample. Data are from one representative out of two independent experiments with three samples per strain each. The mean ± SD is indicated. (**K**) LDH release from TR146 keratinocytes after exposure to the fungal strains for 24 h at 37°C, 5% CO_2_. Each symbol represents one well. (**L–N**) C57BL/6 mice were colonized with *C. albicans* strains 101_*BRG1*^FL/FL^ and 101_*BRG1*^FL/FL^ *CYR1*^E1541K^ (clone 1). (**L**) Tongue fungal burden on day 1 and day 7 after colonization. DL, detection limit. (**M**) PAS-stained histology sections of the tongue on day 1 and day 7 after colonization. (**N**) *ECE1* expression levels in the tongue tissue on day 7 after colonization. Each symbol represents one mouse. Data are pooled from two independent experiments. The mean ± SEM is indicated. Statistical significance was determined using one-way ANOVA (**B–D and I–K**) or unpaired Student’s *t*-test (**E–E, G, L–N**). *P < 0.05, **P < 0.01, ***P < 0.001, ****P < 0.0001. See also Fig. S4 and S5.

Thus, we hypothesized that the *CYR1*^E1541K^ point mutation was indeed responsible for the phenotype of strain 101_*BRG1*^FL^/*BRG1*^FL^(*). In order to assess this hypothesis, we used CRISPR/Cas9 to introduce the mutation directly into the original 101 strain. Modification of one allele was sufficient to drive a strong filamentation phenotype on Spider agar (Fig. 4A) and in contact with TR146 keratinocytes (Fig. 4B; Fig. S4A). Increased *in vitro* filamentation was accompanied by repression of *NRG1* and strong induction of hyphae-associated genes to levels approximating or even exceeding those measured in SC5314 (Fig. 4C; Fig. S4B). Despite strong filamentation and high levels of *ECE1* expression, the 101_*CYR1*^E1541K^ mutant did not phenocopy strain SC5314 regarding damage induction in TR146 keratinocytes (Fig. 4D).

The lack of high damage induction *in vitro* paralleled the inability of the 101_*CYR1*^E1541K^ mutant to drive inflammation *in vivo* in the oral mucosa of experimentally colonized mice (Fig. 4E and F). The morphology of the 101_*CYR1*^E1541K^ mutant in the murine tongue tissue was indistinguishable from that of the parental 101 wild-type strain, although quantification of filament length is impossible on tissue sections (Fig. 4F). Likewise, the *in vivo* expression of hyphae-associated genes by the *CYR1*^E1541K^ mutant in comparison to the parental 101 wild-type strain was not enhanced in the tongue tissue (Fig. 4G). We were unable to test the mutant bearing two copies of the *CYR1*^E1541K^ point mutation *in vivo* because this strain formed aggregates as a result of the hyperfilamentous phenotype, which precluded reproducible colonization of the murine oral mucosa. By day 7, when strain SC5314 is cleared from the experimentally infected tongue (25), fungal counts of the 101_*CYR1*^E1541K^ mutant were still high and indistinguishable from those of the parental wild type strain (Fig. 4E). Fungal elements exhibited a mixed morphology. On very rare occasions, we found larger fungal accumulations at the tongue surface, which in some instances also coincided with small inflammatory foci in the epithelium; this was observed only for the *CYR1*^E1541K^-bearing mutant, but not for the wild-type 101 strain (Fig. 4F). This observation coincided with a small trend toward higher *ECE1* and reduced *NRG1* expression in the tongue tissue (Fig. 4G and Fig. S4C).

We wondered whether the phenotype of 101_*CYR1*^E1541K^ was altered by the presence of two intact *BRG1* alleles, as the *CYR1*^E1541K^-carrying mutant originally emerged from the 101_*BRG1*^FL^/*BRG1*^FL^ strain (Fig. 2G). We therefore introduced the *CYR1*^E1541K^ mutation into this strain to obtain a reconstituted mutant. This resulted in a strain that largely phenocopied 101_*CYR1*^E1541K^. It exhibited strongly enhanced filamentation in both Spider agar and in contact with TR146 keratinocytes, and it strongly expressed hyphae-induced genes *in vitro* (Fig. 4H through J; Fig. S5A and B). However, these changes were not accompanied by enhanced *in vitro* damage induction (Fig. 4K). Likewise, in the oral mucosa of experimentally colonized mice, the *CYR1*^E1541K^ variant did not exert a stronger inflammation-inducing effect when introduced into 101_*BRG1*^FL^/*BRG1*^FL^ than in the wild-type 101 background (Fig. 4L through N; Fig. S5C). We also tested whether the *CYR1*^E1541K^ mutation would render strain 101, carrying one or two intact *BRG1* alleles, more pathogenic when tested in the murine model of systemic candidiasis, which was not the case (Fig. S5D).

Together, the results from these *in vitro* and *in vivo* experiments indicate that the hyperactive variant of Cyr1 indeed has the potential to bypass the repression of some pathogenicity traits under strong hyphae-inducing conditions, such as those used for our *in vitro* assays, but it is insufficient to fully overcome the low-damage-inducing nature of strain 101 under within-host conditions, independently of the functionality of *BRG1*. This also indicates that filamentation and hyphae-associated gene expression under *in vitro* conditions are not good correlates of *C. albicans* pathogenicity *in vivo*.

### The capacity of the hyperactive *CYR1*^E1541K^ point mutation to de-repress filamentation and hyphae-associated gene expression in *C. albicans* is not limited to strain 101

**Fig 5:**
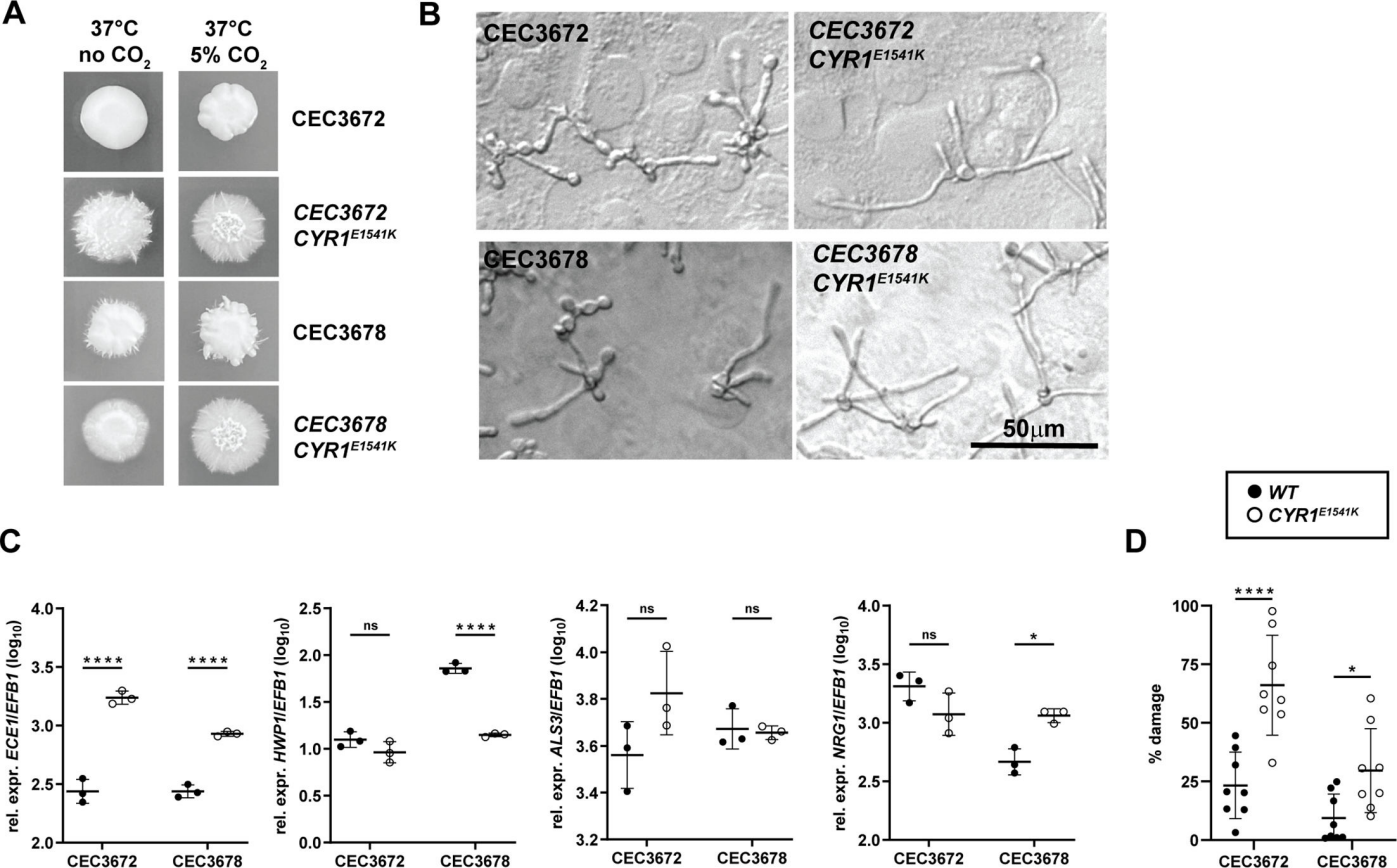
The capacity of the hyperactive *CYR1*^E1541K^ point mutation to de-repress filamentation and hyphae-associated gene expression in low-damage-inducing *C. albicans* is strain-independent. *C. albicans* strains CEC3672, CEC3672_*CYR1*^E1541K^, CEC3678, and CEC3678_*CYR1*^E1541K^ were assessed for their phenotype *in vitro*. (**A**) Colony morphology of the strains grown on Spider agar for 5 days at 37°C. (**B**) Filamentation of the strains put in contact with a monolayer of TR146 keratinocytes for 3.5 h at 37°C, 5% CO_2_. (**C**) *ECE1*, *HWP1*, *ALS3,* and *NRG1* expression levels by the fungal strains after exposure to TR146 keratinocytes for 24 h at 37°C, 5% CO_2_. Each symbol represents one sample. Data are from one representative out of two independent experiments with three samples per strain each. The mean ± SD is indicated. (**D**) LDH release from TR146 keratinocytes after exposure to the fungal strains for 24 h at 37°C, 5% CO_2_. Each symbol represents one well. Data are from one representative out of two independent experiments. The mean ± SD is indicated. Statistical significance was determined using unpaired Student’s *t*-test comparing each mutant with the corresponding parental strain. *P < 0.05, **P < 0.01, ***P < 0.001, ****P < 0.0001.

To obtain further insights into the impact of the strain background on the potential of the *CYR1*^E1541K^ mutation to modulate *C. albicans* phenotype, we turned to other low-damage-inducing isolates that exhibit similar properties in the oral mucosa as strain 101. Strains CEC3672 and CEC3678 (39, 40) both carry two full-length *BRG1* alleles, including the C-terminal extension, encoding Brg1 proteins almost identical to those in the SC5314 genome (Fig. S1). The phenotype of these isolates was comparable to that of strain 101 regarding *in vitro* filamentation, damage induction, release of damage-associated cytokines, and the efficiency to colonize the murine oral mucosa *in vivo* (13). Introducing the *CYR1*^E1541K^ point mutation in these low-damage-inducing isolates rendered them more filamentous, as we previously observed for strain 101, both on Spider agar and when placed in contact with keratinocytes (Fig. 5A and B, compare to Fig. 4A, B, H and I). Likewise, the *in vitro* expression of *ECE1* was enhanced, while the expression of other hyphae-associated genes was less affected than what we observed in the case of strain 101 (Fig. 5C, compare to Fig. 4C and J and Fig. S4B and S5B). Keratinocyte damage was also increased in response to CEC3672 and CEC3678 carrying the hypervirulent *CYR1* variant, compared to the corresponding parental strains, with the degree of the effect being strain-dependent (Fig. 5D, compared to Fig. 4D and K). Together, these data indicate that the Glu-to-Lys mutation in Cyr1 rendered the adenylate cyclase hyperactive (38), which led to de-repression of filamentation and enhanced expression of hyphae-associated genes in multiple normally low-damage-inducing isolates, although the point mutation was insufficient to induce full pathogenicity, as it did not elicit high cellular damage *in vitro* or an inflammatory response at the host interface *in vivo*, as inferred from our results with the 101_*CYR1*^E1541K^ strain.

### The Ras1/cAMP-PKA pathway promotes filamentation in intrinsically low-damage-inducing strains

**Fig 6:**
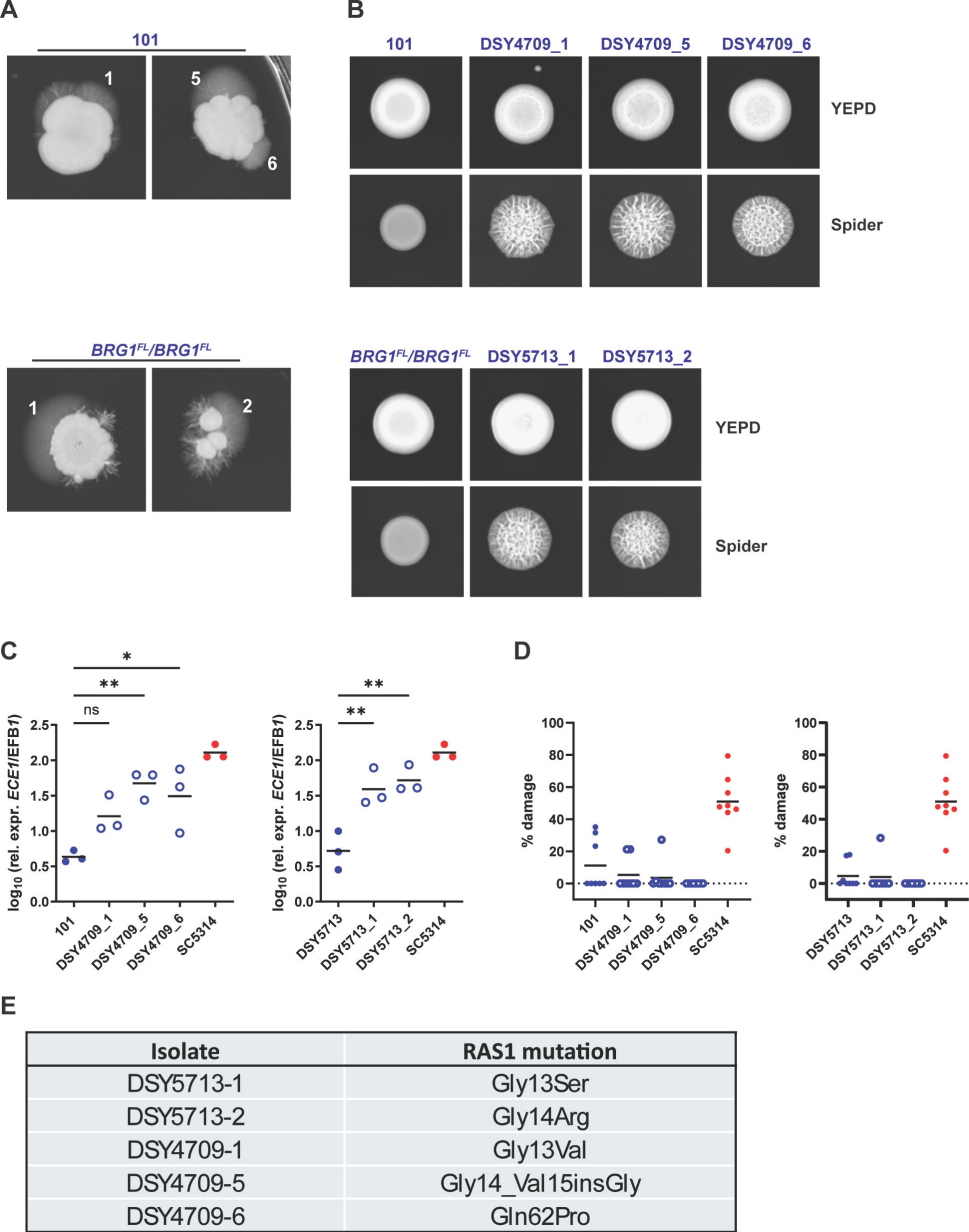
The Ras1-cAMP pathway promotes filamentation in intrinsically low-damage-inducing strains. (**A**) *C. albicans* strain 101 wild type and 101_*BRG1*^FL^/*BRG1*^FL^ were plated on Spider agar for 7 days at 35°C. (**B**) Colony morphology of clones that were isolated from spontaneously outgrown filaments (**A**) when grown on YEPD or Spider agar, as indicated. (**C and D**) *ECE1* expression levels (**C**) and LDH release (**D**) by the clones after exposure to TR146 keratinocytes for 24 h at 37°C, 5% CO_2_. Each symbol represents one sample. The mean ± SD is indicated. Statistical significance was determined using one-way ANOVA comparing each mutant with the corresponding parental strain. *P < 0.05, **P < 0.01; ns, non-significant (*P* > 0.05) (**E**) Mutations in *RAS1* identified in the clones shown in panel **B**.

Although the spontaneously acquired Cyr1^E1541K^ mutation did not explain the highly pathogenic phenotype of strain 101_*BRG1*^FL^/*BRG1*^FL^(*), it has a remarkable capacity to alter the phenotype of several low-damage-inducing isolates *in vitro*. We wondered whether the modulation of the cAMP pathway was a unique event or might occur more frequently. Prolonged growth on Spider agar spawned several filamentation events in both strain 101 and the 101_*BRG1*^FL^/*BRG1*^FL^ mutant (Fig. 6A and B). Clones generated from these spontaneously outgrown colonies confirmed the hyperfilamentous phenotype, which was associated with enhanced expression of *ECE1*, but not sufficient to drive epithelial damage induction (Fig. 6C and D). We then performed whole-genome sequencing of five of the isolated clones to identify mutations in comparison to their parental strains. Interestingly, all identified mutations were in *RAS1* (Fig. 6E; Table S2), a gene encoding a GTPase that mediates Cyr1 activation in response to serum and N-acetylglucosamine stimulation through interaction with the N-terminal Ras association domain of Cyr1 (41). Several of the identified mutations have been previously reported to render *C. albicans* and/or human Ras hyperactive (42–44). Together, the data demonstrate that genes of the cAMP-PKA pathway are hotspots for spontaneously acquired mutations that enhance pathogenicity traits of *C. albicans*. However, as seen with 101-derived strains expressing a hyperactive variant of *Cyr1* (Fig. 4), filamentation associated with mutations in Ras was not sufficient to drive full pathogenicity.

## DISCUSSION

*Candida albicans* is a prominent human pathobiont associated with diseases that vary in severity from mild superficial forms to fatal systemic infections. Despite the pathogenic potential of *C. albicans*, it is, in the first place, a harmless colonizer of mucosal tissues that does not damage the host but instead rather enhances the host resistance to infection (5). The precise mechanisms enabling *C. albicans* to thrive as a resident of the mycobiota remain to be fully clarified. Studying isolates with low intrinsic pathogenicity and their interaction with the host has advanced the understanding of how *C. albicans* establishes colonization and avoids tissue damage and inflammation to preserve homeostasis (13, 14, 25). Here, we explored how the transcription factor Brg1 and the Cyr1 adenylate cyclase modulate pathogenic properties of the *C. albicans* colonizer strain 101. While the introduction of allele variants of the *BRG1* and *CYR1* genes into strain 101, either individually or in combination, increased the strain’s pathogenicity traits *in vitro*, it was not sufficient to increase the strain pathogenicity in the oral mucosal tissue of experimentally colonized mice. These findings underscore the tight restriction of the hyphae-associated gene network in *C. albicans* that guarantees homeostatic colonization in the immunocompetent host.

Defining the properties of *C. albicans* that govern fungal colonization versus inflammation and pathology *in vivo* is the subject of continuous ongoing efforts. Studies with gene-deficient mutant strains provided evidence that filamentation, hyphae-associated gene expression, and cell damage induction are critical pathogenicity traits of *C. albicans* associated with infection and induction of inflammation *in vivo* (45). As such, diverse assays have been developed and are widely used to quantify these fungal properties *in vitro*. Our findings suggest, however, that *in vitro* filamentation and the expression of hyphae-associated genes are not good correlates of *C. albicans* pathogenicity in the oral mucosa. This probably reflects the fact that environmental cues inducing filamentation and the expression of hyphae-associated genes differ between cell culture and inside mammalian tissues. In contrast, the induction of cellular damage in cultured keratinocytes seems to be a more reliable correlate of pathogenicity *in vivo*. This emphasizes that although filamentation is a pre-requisite for pathogenicity, the two processes can be decoupled, and as such, care should be taken when interpreting *in vitro* filamentation assays.

The newly assembled high-quality genomic sequence of strain 101 enabled us to conduct a comparative genome analysis of strains SC5314 and 101, which drew our attention to a rare truncated *BRG1* allele in strain 101. Brg1 regulates the *NRG1* hyphal repressor gene via mRNA destabilization (11) and chromatin remodeling (46). Our previous findings that *NRG1* expression levels are decisive for the low-damage-inducing phenotype of strain 101 (14) led us to explore the functionality of the truncated *BRG1* allele. Indeed, the truncated *BRG1* allele was functionally defective. Replacing the truncated *BRG1* allele of strain 101 with a copy of the functional allele, leading to strain 101 having two full-length, functional alleles, resulted in modest induction of pathogenicity traits *in vitro*; however, this was insufficient to increase pathogenicity in the oral mucosa *in vivo*. These data are consistent with previous reports of allelic differences in individual genes capable of modulating *C. albicans*-host interaction properties (47, 48). The lack of full induction of pathogenicity may, at least in part, be explained by the complex regulation of Brg1 (49, 50). We did only replace the dysfunctional *BRG1* ORF with the full-length *BRG1* ORF from strain 101, without directly modulating the expression levels. This ORF is almost identical to one of the two ORFs encoded by the first *BRG1* allele in SC5314 but differs by several amino acids from that encoded by the second allele. We cannot exclude that the two SC5314 Brg1 proteins have different functionalities that are required for high pathogenicity.

Aiming to explain the acquired high-damage-inducing phenotype of 101_*BRG1^FL^*/*BRG1*^FL^(*), reminiscent of strain SC5314, we identified a gain-of-function mutation in *CYR1*, *CYR1*^E1541K^, previously described to render Cyr1 hyperactive and thereby to promote constitutive filamentation of strain SC5314, even under non-hyphae-inducing conditions (38). In the background of strain 101, the *CYR1*^E1541K^ point mutation led to a strong filamentation phenotype *in vitro* and yet was insufficient to fully de-repress the pathogenicity traits, irrespective of the *BRG1* status of the strain, indicating that other, so far unidentified mutations may also contribute to the conversion of a low- into a high-damage-inducing strain. As a matter of fact, 101_*BRG1^FL^*/*BRG1*^FL^(*) contains, in addition to the Cyr1^E1541K^ point mutation, additional non-synonymous changes in ORFs (Table S1), which may contribute to the phenotype of this isolate. Further investigation of these changes, to identify those that, together with *CYR1*^E1541K^, lead to the high-damage-inducing phenotype of 101_*BRG1^FL^*/*BRG1*^FL^(*), is needed. Pinpointing such change(s) is challenging, as it necessitates extensive characterization of the genomes of strains 101_*BRG1^FL^*/*BRG1*^FL^ and 101_*BRG1^FL^*/*BRG1*^FL^(*), and subsequent evaluation of the validated non-synonymous changes in ORFs using strain engineering. This approach assumes that the phenotypic difference between 101_*BRG1^FL^*/*BRG1*^FL^ and 101_*BRG1^FL^*/*BRG1*^FL^(*) is the consequence of changes in ORFs. Yet, it cannot be excluded that it reflects changes in non-coding regions that are even more challenging to investigate.

We observed a *CYR1*^E1541K^-dependent increase in pathogenicity in other low-damage-inducing strains of *C. albicans*, albeit to variable degrees. While *ECE1* expression was consistently increased, other hyphae-associated genes were not. Thus, the consequences of the *CYR1*^E1541K^ mutation appear to be strain-dependent. This is not unexpected as strain 101 belongs to *C. albicans* clade 2, while the other low-damage-inducing strains belong to *C. albicans* clade 1 (such as strain SC5314). Furthermore, these latter two strains differ by numerous non-synonymous SNPs in filamentation-related genes, such as *HGC1, UME6, and ROB1,* which may influence the penetrance of the *CYR1*^E1541K^ mutation. Strain-specific regulation of the activity of Cyr1 may include the availability of ATP (the substrate for the generation of cAMP), which is regulated by mitochondrial activity, or differential expression of proteins, such as the Pde2 phosphodiesterase (17).

The impact of *CYR1*^E1541K^ on fungal properties differs between *in vitro* and *in vivo* conditions, suggesting that the degree to which the cAMP-PKA pathway is required for filamentation may also differ under *in vivo* and *in vitro* conditions (51). In the mucosal tissue, the threshold to de-repress fungal pathogenicity traits is particularly high and requires an intricate sequence of events that unfold over extended periods of time (40). Cyr1 hyperactivation does not appear strong enough to override the negative regulation of filamentation and hyphae-associated genes by Tup1 and Nrg1 (16). Similarly, the *CYR1*^E1541K^ mutation did not increase the virulence of strain 101 during systemic candidiasis. *C. albicans* is able to colonize different types of host tissue, such as the gut, which presents distinct environmental contexts (1, 45). For instance, bacterial peptidoglycan-like molecules, which are abundant in the intestine, were shown to induce filamentation by directly binding to Cyr1 (52). Therefore, we cannot exclude that Cyr1 hyperactivation may affect *C. albicans* phenotype in the gut.

In addition to the initially identified spontaneous highly damage-inducing mutant 101_*BRG1^FL^*/*BRG1*^FL^ (*), we recovered additional spontaneous mutants outgrowing from strain 101, all of which exhibited mutations in the N-terminal part of the *RAS1* gene known to freeze the protein in a permanently active state (53, 54). Ras1 acts upstream of Cyr1 to induce filamentation (17). In addition, Ras1 can also induce filamentation independently of Cyr1 by activating Cdc24, leading to activation of the Cph1 transcription factor involved in the filamentation program (55). The observation that all spontaneously developed variants exhibited mutations affecting the cAMP-PKA pathway raises important questions regarding the driving force inducing these mutations. An environmental cue specific to the conditions under which the fungus was cultured may specifically target the cAMP-PKA pathway, or the cAMP-PKA signaling cascade may serve as a preferential target due to its central role in facilitating environmental adaptation without significantly jeopardizing the fungus’s survival.

Together, we demonstrate that strain 101 tightly regulates fungal pathogenicity traits. Additional allele variants may contribute to the inherently low-damage-inducing properties, and replacing a single gene turned out to be insufficient to overcome the strain-intrinsic colonizer program, which supports the maintenance of homeostasis in the colonized mucosal tissue. In addition to the strain-intrinsic properties of a strain, the functional state of a strain also depends on the environmental conditions. When exposed to hyphae-inducing conditions, such as those characteristic of the macrophage phagosome or in the epithelium of the IL-17-deficient host, even low-damage-inducing strains can gradually express increased pathogenicity features (40, 56–58). We observed a similar phenomenon when growing strain 101 on Spider agar, thereby further demonstrating the strong adaptability of *C. albicans* to its environment.

## MATERIALS AND METHODS

### Fungal strains

Strains used in this study are listed in Table 1. All strains were maintained on YPD agar for short term and in glycerol-supplemented medium at −80°C for long-term storage. Cultures were inoculated at OD_600_ = 0.1 in YPD medium (using a pre-culture to adjust the OD_600_) and grown at 30°C and 180 rpm for 15–18 h. At the end of the culture period, yeast cells were washed in PBS, and their concentration was determined by spectrophotometry, whereby 1 OD_600_ = 10^7^ yeast cells.

**TABLE 1:** *C. albicans* strains used in this study^*a*^

Strain	Collection #	Genotype	Parental	Reference
SC5314	CA1	Clinical isolate	n/a	(57)
101	DSY4709 CA117	Clinical isolate	n/a	(25)
CEC3672	CA327	Clinical isolate	n/a	(39)
CEC3678	CA328	Clinical isolate	n/a	(39)
SC5314_*brg1*Δ/Δ	DSY5875 CA329	*arg4*∆/*arg4*∆, *leu2*∆/*leu2*∆, *his1*∆/*his1*∆, *URA3*/*ura3*∆::*imm^434^*, *IRO1*/*iro1*∆::*imm^434^*, *brg1*∆::*HIS1*, *brg1*∆::*LEU2*	SN152	(34)
SC5314_BRG1/BRG1	DSY5876 CA330	*arg4*∆/*arg4*∆, *leu2*∆/*leu2*∆::*LEU2*, *his1*∆/*his1*∆::*HIS1*, *URA3*/ *ura3*∆::*imm^434^*, *IRO1*/*iro1∆*::*imm^434^*	SN152	(34)
SC5314_*brg1*∆/*BRG1*^FL^	DSY5877 CA331	*arg4*∆/*arg4*∆, *leu2*∆/*leu2*∆, *his1*∆/*his1*∆, *URA3*/ *ura3*∆::*imm^434^*, *IRO1*/*iro1∆*::*imm^43^*, *brg1*∆::*HIS1*::*BRG1*^FL^::*NAT1*, *brg1*∆::*LEU2* (FL allele of strain 101*)*	DSY5875	This study
SC5314_brg1∆/BRG1^TRUNC^	DSY5880 CA334	*arg4*∆/*arg4*∆, *leu2*∆/*leu2*∆, *his1*∆/*his1*∆, *URA3*/*ura3*∆, *IRO1*/*iro1*∆, *brg1*∆::*HIS1*::*BRG1*^TRUNC^::*NAT1*, *brg1*∆::*LEU2* (TRUNC allele of strain 101)	DSY5875	This study
SC5314_*BRG1*/*BRG1*^TRUNC^	DSY5720 CA273	*BRG1*/ *BRG1*^TRUNC^ *::NAT1*	SC5314	This study
101_*BRG1*^FL^/*BRG1*^TRUNC^	DSY5710 CA267	*BRG1*^FL^/*BRG1*^TRUNC^::*NAT1*	101	This study
101_*BRG1*^FL^/*BRG1*^FL^	DSY5713 CA270	*BRG1*^FL^/*BRG1*^FL^::*NAT1*	101	This study
101_*BRG1*^FL^/*BRG1*^FL^(*)	DSY5722 CA271	*BRG1*^FL^/*BRG1*^FL^::*NAT1*, *CYR1*/*CYR1*^E1541K^	DSY5713	This study
101_*CYR1*^E1541K^	DSY5731 CA279	*CYR1*/*CYR1*^E1541K^, *ENO1*::pV1025	101	This study
101_*CYR1*^E1541K^/*CYR1*^E1541K^ (#1)	DSY5730 CA295	*CYR1*^E1541K^/*CYR1*^E1541K^, *ENO1*::pV1025	DSY5731	This study
101_*CYR1*^E1541K^/*CYR1*^E1541K^ (#2)	DSY5732 CA296	*CYR1*^E1541K^/*CYR1*^E1541K^, *ENO1*::pV1025	DSY5731	This study
101_*BRG1*^FL^/*BRG1*^FL^ *CYR1*^E1541K^ (#1)	DSY5862 CA335	*BRG1*^FL^/*BRG1*^FL^::*NAT1*, *CYR1*^E1541K^, *ENO1*::pDS2168	DSY5713	This study
101_*BRG1*^FL^/*BRG1*^FL^ *CYR1*^E1541K^ (#2)	DSY5863 CA336	*BRG1*^FL^/*BRG1*^FL^::*NAT1*, *CYR1*^E1541K^, *ENO1*::pDS2168	DSY5713	This study
CEC3672_*CYR1*^E1541K^	DSY5791 CA299	*CYR1*/*CYR1*^E1541K^ , *ENO1*::pV1025	CEC3672	This study
CEC3678_*CYR1*^E1541K^	DSY5774 CAC303	*CYR1*/*CYR1*^E1541K^, *ENO1*::pV1025	CEC3678	This study
DSY4709_1 (*)	DSY5840	*RAS1/RAS1^G14R^*	101	This study
DSY4709_5 (*)	DSY5842	*RAS1/RAS1^G13S^*	101	This study
DSY4709_6 (*)	DSY5844	*RAS1/RAS1^G14V^*	101	This study
DSY5713_1 (*)	DSY5846	*BRG1*^FL^/*BRG1*^FL^::*NAT1, RAS1/RAS1^G14V15insG^*	DSY5713	This study
DSY5713_2 (*)	DSY5848	*BRG1*^FL^/*BRG1*^FL^::*NAT1, RAS1/RAS1^Q62P^*	DSY5713	This study

^*a*^ n/a, not available.

### Identification of the truncated *BRG1* allele

PCR of *BRG1* alleles on strain 101 was accomplished using primers BRG1-5 and BRG1-3 (Table 2) and analyzed by gel electrophoresis on 1% agarose.

**TABLE 2:** Primer list

Primer	Sequence
BRG1-5	TACATTATAAATATTCACTACTATTCCAGAAAT
BRG1-3	AATACCGCCAGCTAACATATGTTG
BRG1-P1	CTTTCTTAATATCACACAAAAAGTGTTTATTCTTC
BRG1-NAT1-5R	GTATTCTGGGCCTCCATGTCGCATTAACAAGTGTTGATTA
BRG1-NAT1-5F	TAATCAACACTTGTTAATGCGACATGGAGGCCCAGAATAC
BRG1-NAT1-3R	GTATTCTGGGCCTCCATGTCGCATTAACAAGTGTTGATTA
BRG1-NAT1-3F	GAATGCTGGTCGCTATACTGAGTGTAATTAGTTTTTTCCT
BRG1-P2	CTCTTAACTTACCACACAAAAAATGCTG
BRG1-P3B	CCATTTAATGCATTTAACCCGTTTAACATTT
BRG1-P4	CATTATTTGTCCTGGACGTCGTCT
SNR52/N	GCGGCCGCAAGTGATTAGACT
sgRNA/N	GCAGCTCAGTGATTAAGAGTAAAGATGG
SNR52/F	AAGAAAGAAAGAAAACCAGGAGTGAA
Cyr1_guide Rev	CTTCAATTTACGCTCACCAACAAATTAAAAATAGTTTACGCAAGTC
sgRNA/R	ACAAATATTTAAACTCGGGACCTGG
Cyr1_guide For	TTGGTGAGCGTAAATTGAAGGTTTTAGAGCTAGAAATAGCAAGTTAAA
Cyr1-A	TGACAATTGAACGAGAATTGAATGCATTGGAGGATCTTGGGTGTAACTATTTTAAAATTGGTGAGC GTAAATTGAAGGGATTGA
Cyr1-B	AAATATATCATATCTCAATTTCAATCTATTGGTAAACACCAAAGTGATAGGTTCTGGTGTTTTCAATCCCTT CAATTTACGCTC
HYGR-NotI	GCTTAAAAGCGGCCGCATTTTATGATGGAATGAATGGGATGAAT
HYGR-NheI	GCGCAAAGCTAGCGTATAGTGCTTGCTGTTCGATATTG

### Mutant generation

#### *BRG1*^TRUNC^ and *BRG1*^FL^ revertants in the SC5314 background

*BRG1* revertants were constructed with a *brg1*∆/∆ mutant available from the Homann mutant collection (DSY5875) (34). A PCR fusion strategy was used to amplify *BRG1* alleles fused to the *NAT1* selection marker. The *BRG1* alleles with a 500 bp promoter from strain 101 were first amplified with primers BRG1-P1 and BRG1-NAT1-5R (overlapping primer with *NAT1*). Next, the *NAT1* marker was amplified from pJK795 with BRG1-NAT1-5F and BRG1-NAT1-3R. Finally, the *BRG1* downstream sequence (500 bp) was amplified with primers BRG1-P2 and BRG1-NAT1-3F (overlapping primer with *NAT1*). After PCR fragment purification, equimolar amounts of the three fragments were used with nested primers BRG1-P3B and BRG1-P4 in a fusion PCR to result in a 4 kb fragment. This fragment was used to transform DSY5875 in order to replace the deleted *BRG1* alleles with 101 *BRG1* alleles. This was achieved by an RNA-protein complexes (RNPs) approach, as reported in Grahl et al. (59), which employs reconstituted, purified Cas9 protein in complex with a scaffold and gene-specific guide RNAs. A gRNA specific for the *BRG1* promoter (TGGGTGTAGAGAAACGATGT), upstream of the fusion PCR construct, was designed *in silico* using Geneious Prime and obtained from IDT (Integrated DNA Technologies, Inc.) as CRISPR guide RNA (crRNA), containing 20 bp homologous to the target gene fused to the scaffold sequence. RNPs were created using the Alt-R CRISPR-Cas9 system from IDT. Briefly, the *BRG1* crRNA and tracrRNA (a universal transactivating CRISPR RNA) were first dissolved in RNase-free distilled water (dH_2_O) at 100 µM and stored at −80°C. The complete guide RNA was generated by mixing equimolar concentrations (4 µM final) of the gene-specific crRNA and tracrRNA to obtain a final volume of 3.6 µL per transformation. The mix was incubated at 95°C for 5 min and cooled down to room temperature. The Cas9 nuclease 3NLS (60 µM stock from IDT) was diluted to 4 µM in dH_2_O at a volume of 3 µL per transformation. RNPs were assembled by mixing guide RNAs (3.6 µL of gene-specific crRNA/tracrRNA) with 3 µL of diluted Cas9 protein, followed by incubation at room temperature for 5 min. Transformation of *C. albicans* was carried out by electroporation using 6.6 µL of gene-specific RNPs, 40 µL of *C. albicans* cells, and 1–2 µg of the *BRG1* fusion PCR (up to 3.4 µL volume). Transformants were selected on YEPD plates with nourseothricin (200 µg/mL) for 3 days at 35°C. After selection of transformants, identification of the specific 101 *BRG1* alleles introduced in DSY5875 was verified by targeted sequencing analysis. DSY5877 and DSY5880 contained the *BRG1*^FL^ and *BRG1*^TRUNC^ alleles, respectively, in which the *HIS1* selective marker in one *brg1*∆ allele of DSY5875 was replaced by *NAT1.*

A wild-type *BRG1* allele was also replaced by *BRG1*^TRUNC^ allele in SC5413. For this, the construction of the repair fragment followed the above-mentioned fusion PCR approach. Two guides specific for *BRG1* in SC5314, including 5-BRG1-guide-SC (TGGGAAAGAAGAGATTTACA) and 3-BRG1-guide-SC (TTTAAAAAGCATCAATCAAT), were used in the RNP complexes construction. A minor adaptation was that the RNPs were concentrated by 2-fold and mixed each (3.3 µL) with the *BRG1* repair fragment for electroporation. Transformants were analyzed by targeted sequence analysis, which yielded DSY5720.

#### *BRG1*^TRUNC^ and *BRG1*^FL^ revertants in the 101 background

To replace specific *BRG1* alleles in the 101 background, two different guide fusions were designed, including 5-BRG1-guide-mut-allele (TATTTATAATGTAACAAGTA) and 3-BRG1-guide-mut-allele (CTGCACTGAGATATAAAAAG), which were used in the RNP complexes. The RNPs were concentrated by 2-fold and mixed each (3.3 µL) with the *BRG1* repair fragment for electroporation. Transformants were analyzed by targeted sequence analysis, yielding DSY5710 and DSY5713, which contain the *BRG1*^FL^/*BRG1*^TRUNC^ alleles and *BRG1*^FL^/*BRG1*^FL^ alleles, respectively.

#### Generation of Cyr1^E1541K^ mutants

The introduction of the E1541K mutation in Cyr1 was achieved with a transient guide RNA expression following published protocols (60). First, the guide expression was constructed using two overlapping PCRs obtained with the primer pairs SNR52/F-Cyr1_guide Rev and sgRNA/R-Cyr1_guide, using pV1093 (61) as a template. Fusion PCR between the two fragments was performed with primers SNR52/N and sgRNA/N. Next, the repair fragment (containing the Cyr1^E1541K^ mutation without protospacer adjacent motif (PAM) sites) was obtained by mixing primers Cyr1-A and Cyr1-B, followed by self-priming and 20 PCR cycles. The two PCRs were then mixed with pV1025 previously digested by *Sac*I and *Kpn*I to transform strains 101, CEC3672, and CEC3678 (14) by electroporation. Transformants were selected on YEPD plates with nourseothricin (200 µg/mL) for 3 days at 35°C. Targeted sequencing of transformants yielded heterozygous (DSY5731, DSY5791, DSY5774) and homozygous (DSY5730, DSY5732) Cyr1^E1541K^ mutants.

In order to introduce the E1541K mutation in the background of isolate DSY5713, pV1025 was first modified by replacing *SAT1* with *HYGR* (hygromycin resistance). This was achieved by PCR amplification of *HYGR* from PYM70 (62) using primers HYGR-NotI and HYGR-NheI, followed by insertion into pV1025 at the *Not*I and *Nhe*I restriction sites, yielding pDS2168. This plasmid was digested by *Kpn*I and *Eco*RV in the transformation procedure described above. After the selection of transformants, targeted sequencing yielded heterozygous Cyr1^E1541K^ mutants DSY5862 and DSY5863.

### Genome assembly and annotation of strain 101

High-quality genomic DNA was prepared according to published procedures (63). High molecular weight DNA was sheared with Megaruptor (Diagenode, Denville, NJ, USA) to obtain 10–15 kb fragments. After shearing, the DNA size distribution was checked using a Fragment Analyzer (Advanced Analytical Technologies, Ames, IA, USA). The sheared DNA (1.8 µg) was used to prepare a SMRTbell library with the PacBio SMRTbell Express Template Prep Kit 2.0 (Pacific Biosciences, Menlo Park, CA, USA), according to the manufacturer’s recommendations. The resulting library was size-selected on a Blue Pippin system (Sage Science, Inc. Beverly, MA, USA) for molecules larger than 6 kb. It was sequenced with v2.2/v2.0 chemistry and adaptive loading on a PacBio Sequel II instrument (Pacific Biosciences, Menlo Park, CA, USA) with a 30-hour movie length and 2-hour pre-extension time using one SMRT Cell 8M. Genome assembly was conducted with hifiasm (v0.16.1-r375) (64), and the resulting contigs were benchmarked against the *C. albicans* SC5314 reference (A22-s07-m01-r145) using MUMmer (v3.0) (65), QUAST (66), and *BUSCO* (v5.4.2) (67) to assess completeness, contiguity, and gene content. *Ab initio* gene prediction was performed with AUGUSTUS (v3.4.0) (68), and annotations were refined by mapping to closest orthologs from the OMA Knowledgebase using OMA Fast Mapping (69). Protein-coding gene models underwent manual curation in Geneious Prime (https://www.geneious.com/) to resolve ambiguities and validate exon–intron boundaries. Non-coding RNA loci were identified with Infernal (v1.1.4) (70) against the Rfam database (v14.8) (67). Genome feature files (GFF) and sequence files (FASTA) were manipulated and formatted using EMBOSS (v6.6.0) (71) and integrated into the final annotation with MAKER (v2.31.9) (72). The complete diploid assembly and raw sequencing reads are available under NCBI BioProject PRJNA923600.

### Genome-wide mutant analysis

Spontaneous filamenting mutants were subjected to genome sequence analysis. Genomic DNA from isolates DSY5722, DSY5846, and DSY5848 (DSY5713 as parent) and from isolates DSY5840, DSY5842, and DSY5844 (101 as parent) was first obtained by a spheroplasting method (73). Genomic libraries were prepared from 100 ng of DNA using the Illumina DNA Prep Kit (Illumina) and Unique Dual Indexed oligonucleotides (IDT). PCR amplification was performed for five cycles. Libraries were quantified by a fluorimetric method, and their quality was assessed on a Fragment Analyzer (Agilent Technologies). Sequencing was performed as a 2 × 150-cycle paired-end run either on a MiSeq v2 flow cell (DSY5713 and DSY5722) or on a NovaSeq 6000 v1.5 flow cell (Illumina) (DSY5846, DSY5848, DSY5840, DSY5842, and DSY5844). Sequencing data were demultiplexed using the bcl2fastq2 Conversion Software (v. 2.20, Illumina). Data can be obtained under Bioproject PRJNA1147312.

Single nucleotide polymorphism (SNP) analysis was conducted to compare the isolates DSY5713 and spontaneous filamenting mutant DSY5722. As in Ropars et al. (24), each set of paired-end reads was mapped against the *C. albicans* reference genome SC5314 haplotype A (version A22-s07-m01-r130), downloaded from the *Candida* Genome Database (http://www.candidagenome.org), and SNPs were called using Genome Analysis Toolkit version 3.6. We created a table encompassing SNPs across the DSY5713 and DSY5722 isolates (Table S1).

SNP analysis from isolates DSY5846, DSY5848, DSY5840, DSY5842, and DSY5844 was performed with CLC Genomics Workbench (v24) using the variant detection tools and the annotated genome from isolate 101. After read mapping to the 101 genome, variants differing from those present in DSY5713 and DSY5722 were filtered using the variant filtering tool in CLC Genomics Workbench (Table S2).

#### Growth curve assays

*C. albicans* was inoculated at OD_600_ = 0.1 in 200 μL YPD or F12 cell culture medium (see below) in a 96-well plate and grown in Synergy H1 plate reader (Bio Tek) for 24 h at 30°C with double orbital shaking (3 mm amplitude) and OD_600_ measurements every 15 min, each preceded by a 10-second linear shaking pulse (1 mm amplitude).

#### Colony morphology assays

For assessing filamentation on Spider agar (10 g D-mannitol, Sigma, Cat. no. M4125; 10 g nutrient broth no.1, Sigma, Cat. no. 70122; 2 g K_2_HPO_4_, Sigma, Cat. no. 795496; and 13.5 g agar, Sigma, Cat. no. A1296 per 1 L H_2_O), 3 µL of a yeast cell suspension at 10^5^ cells/mL were spotted, and plates were incubated for specific timing and temperatures as indicated in the figure legends. In some experiments, plates were incubated in the presence of 5% CO_2_. Filamentation was assessed on YEPD agar (yeast extract peptone dextrose [YEPD], 1% Bacto peptone [Difco Laboratories, Basel, Switzerland], 0.5% yeast extract [Difco], 2% glucose [Sigma], and 2% agar [Difco]) and YCB/BSA agar (1.17% yeast carbon base [Becton Dickinson] and 2% BSA [Sigma]). Overnight cultures in YEPB were diluted in water to obtain single colonies. Plates were incubated at 35°C for 5 to 7 days.

#### Keratinocyte cell culture

The human oral keratinocyte cell line TR146 (74) was grown in DMEM medium (Sigma, Cat. no. D5796) supplemented with 10% FCS, 1% penicillin, and 1% streptomycin at 37°C and 5% CO_2_. For RNA isolation and filamentation assay, cells were seeded at 1 × 10^5^ cells/well in 24-well tissue culture plates and for cell damage assay at 4 × 10^4^ cells/well in 96-well tissue culture plates, respectively, and grown to confluent monolayers for 2 days prior to infection, as described below for the individual assays. One day prior to the experiment, the DMEM medium was replaced by F12 medium (Hams’s nutrient mixture F12 medium (Gibco, Cat. no. 21765029) supplemented with 1% FCS).

#### Filamentation assay

Monolayers of TR146 cells in 24-well tissue culture plates, prepared as described above, were infected with 5 × 10^4^ yeast cells per well in a 24-well plate and incubated for 3.5 h at 37°C and 5% CO_2_. Cells were fixed in 2% PFA for 20 min at 4°C. The PFA was then exchanged by PBS for imaging with an EVOS FL Auto microscope (Life Technologies). Filament length was determined with Image J. A total of 20–30 filaments were analyzed per imaged fields, with 10 fields imaged per well and two wells analyzed per strain.

#### Cell damage assay

Damage induction in TR146 cells by *C. albicans* was performed as described (75). Briefly, cell monolayers in 96-well tissue culture plates prepared as described above were infected with 2 × 10^4^ yeast cells per well and incubated for 24 h at 37°C and 5% CO_2_. Control wells were incubated with medium only or with 1% Triton-X-100 to determine 100% damage. LDH release into the supernatant was quantified with the LDH cytotoxicity kit (Roche) according to the manufacturer’s instructions.

#### RNA isolation from infected keratinocyte cultures

Monolayers of TR146 cells in 24-well tissue culture plates prepared as described above were infected with 1 ×10^5^ yeast cells per well. After 24 h of incubation at 37°C and 5% CO_2_, the medium was removed, cells were frozen on liquid nitrogen, and stored at −80°C until further processing. RNA was isolated with the RNeasy Mini Kit (Qiagen). Briefly, the cells were lysed in RLT lysis buffer and homogenized with 0.5 mm glass beads (Sigma, Cat. no. G1277) using a Tissue Lyzer (Qiagen) seven times for 2 min at 30 Hz, with 30-second cooling periods on ice between cycles. Cell debris was removed by centrifugation. One volume of 75% ethanol was admixed, and each sample was transferred to an RNeasy spin column. The loaded columns were washed using RW1 buffer and RPE buffer. RNA was eluted in 30 μL RNase-free water.

#### Animals

WT C57BL/6j mice were purchased by Janvier Elevage and kept in specific pathogen-free conditions at the Institute of Laboratory Animals Science (LASC, University of Zurich, Zurich, Switzerland). Female mice were used at 8–14 weeks in age-matched groups. Infected and uninfected animals were kept separately to avoid cross-contamination.

#### Oral colonization of mice with *C. albicans*

Mice were infected sublingually with 2.5 × 10^6^ *C. albicans* yeast cells as described (76), without immunosuppression. In brief, mice were anesthetized by injection of 100 mg/kg ketamine and 20 mg/kg xylazine in sterile saline i.p. administered in three doses. *C. albicans* was administered by depositing a 2.5 mg cotton ball that was soaked in 100 µL suspension at 5 × 10^7^ yeast cells/mL under the tongue for 80–90 min. Mice were kept on a heating mat at 35–37°C during the entire period of anesthesia, administered with 10 mL/kg sterile saline to stabilize the circulation, and vitamin A ointment was applied to avoid drying out of the eyes.

#### Systemic infection of mice with *C. albicans*

Mice were infected by intravenous injection of 2.5 × 10^5^ *C. albicans* yeast cells for 3 days.

#### Determination of fungal burden

For determination of the fungal burden, the tongue (or kidney) of euthanized animals was removed and, homogenized in sterile 0.05% NP40 in H_2_O for 3 min at 25 Hz using a Tissue Lyzer (Qiagen), and serial dilutions were plated on YPD agar containing 100 µg/mL ampicillin.

#### Histology

Tongue tissue was fixed in 4% PBS-buffered paraformaldehyde overnight and embedded in paraffin. Sagittal sections (9 µm) were stained with periodic-acidic Schiff (PAS) reagent, counterstained with hematoxylin, and mounted with Pertex (Biosystem) according to standard protocols. Images were acquired with a digital slide scanner (NanoZoomer 2.0-HT, Hamamatsu) and analyzed with NDP.view2.

#### RNA isolation from colonized tongue tissue

Isolation of total RNA (including fungal RNA) from murine tongue was performed using TRI reagent (Sigma-Aldrich) according to the manufacturer’s protocol. Prior to the addition of chloroform, the tongue tissue was homogenized in the presence of a steel ball for 3 min at 25 Hz, followed by homogenization with 0.5 mm glass beads (Sigma) twice for 2 min at 30 Hz, with a 30-second cooling period between cycles. Both homogenizations were performed using a Tissue Lyzer (Qiagen).

#### RT-qPCR

cDNA was generated using RevertAid reverse transcriptase (ThermoFisher Scientific, Cat. no. EP0452). Quantitative PCR was performed with SYBR Green (Roche, Cat. no. 4913914001) on a QuantStudio 7 Flex instrument (Life Technologies). The primers used in this study are listed in Table 3. All qPCR reactions were performed in duplicates, and the relative expression (rel. expr.) of each gene was determined after normalization to *EFB1* or *ACT1* housekeeping gene transcript levels.

**TABLE 3:** Primers used in this study for detecting *C. albicans* transcripts^*a*^

Gene	Forward primer (5′ → 3′)	Reverse primer (5′→ 3′)
*EFB1*	CATTGATGGTACTACTGCCAC	TTTACCGGCTGGCAAGTCTT
*ACT1*	TGCTGAACGTATGCAAAAGG	TGAACAATGGATGGACCAGA
*BRG1*	AATCAACCATCACACCCTGC	AATGGCCTGGATGTTGATGC
*NRG1*	AACCTCAGCCATACCATCAAC	GTAATTAGCCCTGGAGATGGTC
*ECE1*	CCAAAATTGCCTGTGCTACTG	CTCTTCATGTTGAATTCTGGAGC
*ALS3*	GGTCTCAATCCTATACCACTGC	GGTTGGTGTAATGAGGACGAG
*HWP1*	CGGAATCTAGTGCTGTCGTCTCT	CCTTCAAATGTAGAAATAGGAGCAAC

^*a*^ All primers for *C. albicans *transcripts were designed to bind to sequences that are conserved between isolates SC5314 and 101.

## Data Availability

All raw data linked to this study are publicly available on Zenodo (doi: 10.5281/zenodo.19075336). The complete diploid assembly and raw sequencing reads for strain 101 are available under NCBI BioProject PRJNA923600. Sequencing data for strains DSY5846, DSY5848, DSY5840, DSY5842 and DSY5844 are available under under BioProject PRJNA1147312 (under “SRA experiments”).

## SUPPLEMENTAL MATERIAL

Supplemental material mbio.00415-26-s0001.docx Supplemental figures and captions for supplemental tables.

Table S1 mbio.00415-26-s0002.xlsx Genome comparison of strains.

Table S2 mbio.00415-26-s0003.xlsx Spontaneous mutations.

ASM does not own the copyrights to Supplemental Material that may be linked to, or accessed through, an article. The authors have granted ASM a non-exclusive, world-wide license to publish the Supplemental Material files. Please contact the corresponding author directly for reuse.
