# Supplementary material for: Manipulation of regulators of morphogenesis is not sufficient to render a Candida albicans colonizer strain pathogenic
Source: mBio. 2026 Apr 7;17(5):e00415-26. doi: 10.1128/mbio.00415-26 (PMC13170366; doi:10.1128/mbio.00415-26)
Supplement: Supplemental material — Supplemental figures and captions for supplemental tables. [file mbio.00415-26-s0001.docx]

**Supplementary FigureS and Legends**


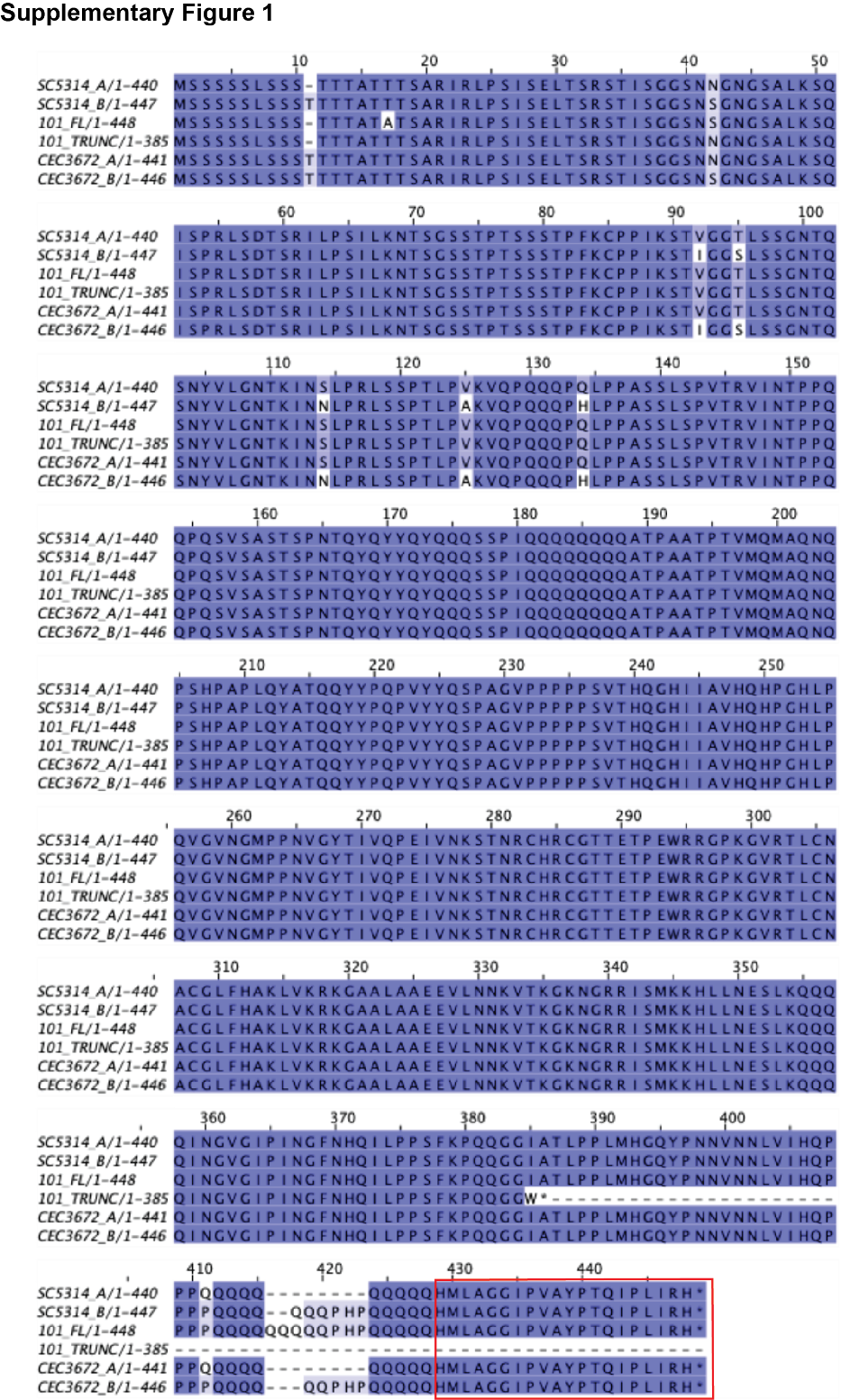


**Figure S1 (related to Figure 1). Alignment of Brg1 proteins from *C. albicans* strains SC5314, 101 and CEC3672**. For strains SC5314 and CEC3672, *BRG1* haplotypes were inferred from SNP data obtained for 182 genome-sequenced strains (24) using PHASE 2.0 as previously described (83). Haplotypes were subsequently edited to consider insertion and deletion events identified using GATK (24). For strain 101, *BRG1* haplotypes were obtained based on the genome assembly produced in this study. The deduced amino acid sequences for the 6 *BRG1* haplotypes were aligned using MUSCLE (84) and colored using JalView (85). The level of conservation of the amino acids across the 6 sequences is shown with a descending coloring ranging from dark blue to white. The amino acid C-terminal extension shared by all Brg1 proteins and lacking in the Brg1 protein deduced from Assembly 22 of the *C. albicans* SC5314 genome is boxed in red. Data obtained for strain CEC3678 are not shown as they are identical to those obtained for strain CEC3672.

**
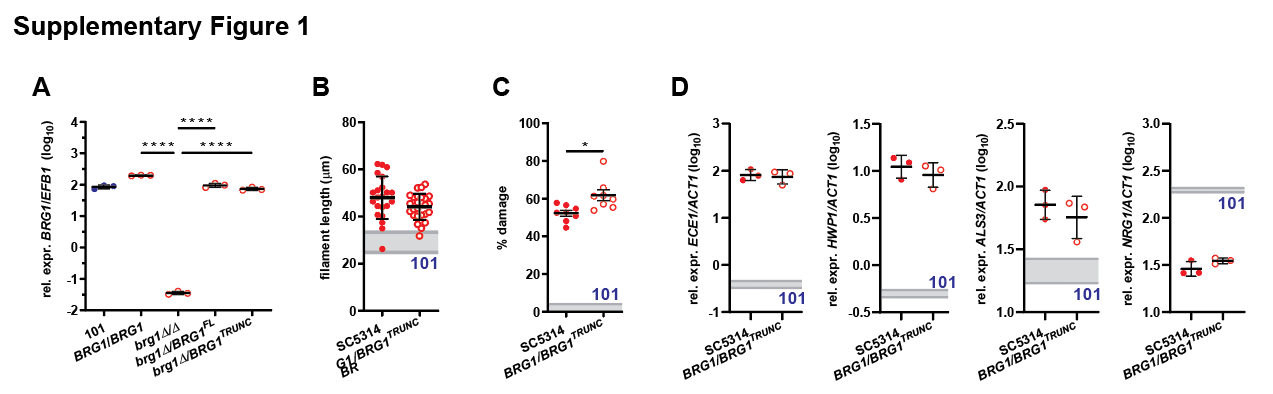
**

**Figure S2 (related to Figure 1). The truncated *BRG1* allele identified in strain 101 is unable to drive pathogenicity in strain SC5314.** **A.** *BRG1* expression levels in *C. albicans* strains 101 wild type, SC5314_*BRG1*/*BRG1*, SC5314_*brg1*Δ/Δ, SC5314_*brg1*∆/*BRG1*^FL^, SC5314_ *brg1*∆/*BRG1*^TRUNC^ after exposure to TR146 keratinocytes for 24 hours at 37°C, 5% CO_2_. Each symbol represents one sample. Data are from one representative out of two independent experiments with three samples per strain each. The mean ± SD is indicated. **B.-D.** *C. albicans* strains SC5314 wild type and SC5314_*BRG1*/*BRG1*^TRUNC^ were assessed for their phenotype *in vitro*. **B.** Quantification of filament length of strain put in contact with TR146 cells. Each symbol represents the mean filament length of 20 – 30 filaments per visual field. Data are pooled from two independent experiments with 10-12 visual fields analyzed per strain each. The mean ± SD is indicated. **C.** LDH release from TR146 keratinocytes after exposure to the fungal strains for 24 hours at 37°C, 5% CO_2_. Each symbol represents one well. Data are pooled from two independent experiments with 4 wells per strain each. The mean ± SEM is indicated. **D.** *ECE1*, *HWP1*, *ALS3* and *NRG1* expression levels by the fungal strains after exposure to TR146 keratinocytes for 24 hours at 37°C, 5% CO_2_. Each symbol represents one sample. Data are from one representative out of two independent experiments with three samples per strain each. The mean is indicated. Statistical significance was determined using one-way ANOVA (C) or student’s *t*-test (D-F). ***p<0.001, ****p<0.0001.

**
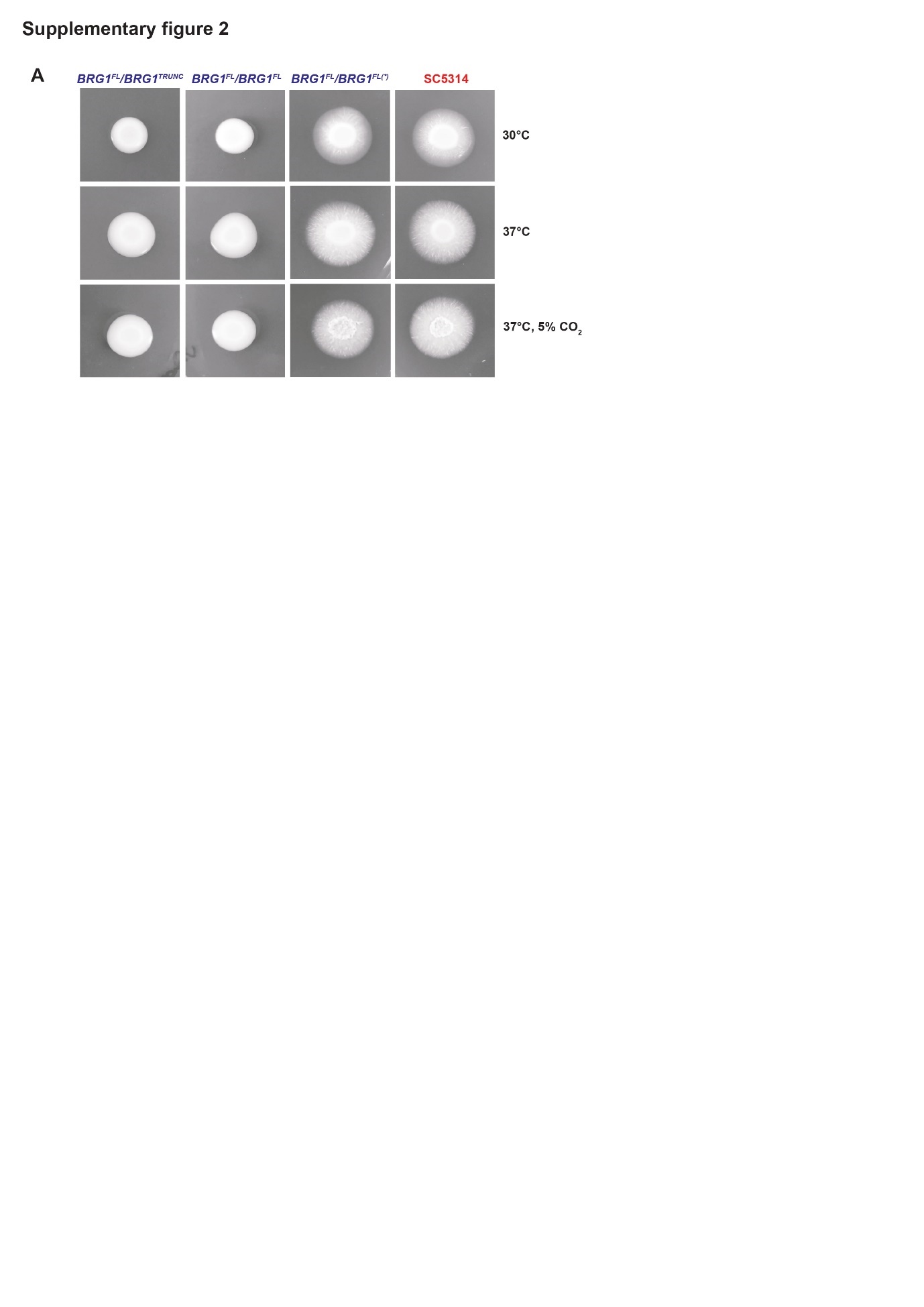
**

**Figure S3 (related to Figure 2).** **Restoring the full-length *BRG1* allele in strain 101 is insufficient to increase the strain’s filamentation.** Colony morphology of *C. albicans* strains 101_*BRG1*^FL^/*BRG1*^TRUNC^, 101_*BRG1*^FL^/*BRG1*^FL^, 101_*BRG1*^FL^/*BRG1*^FL^(*), and SC5314 wild type grown on Spider agar for 5 days under the indicated conditions**.**

**
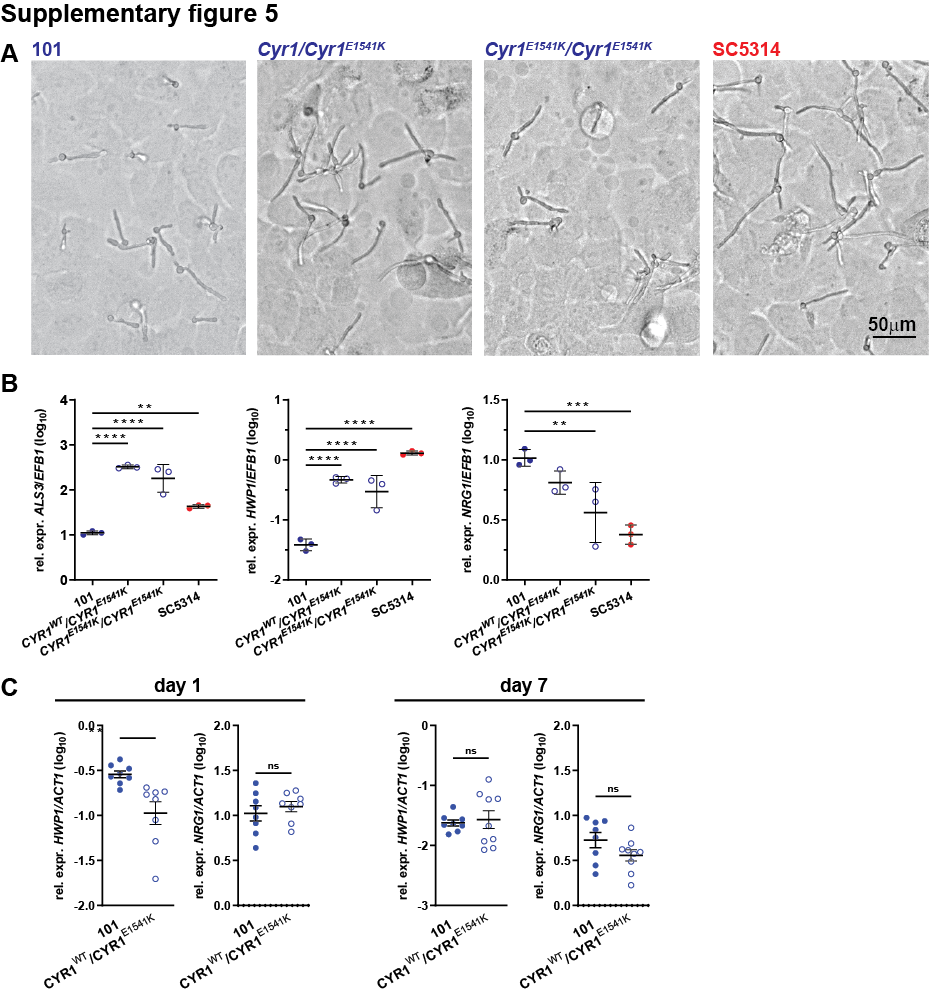
**

**Figure S4 (related to Figure 4).** ***CYR1*^E1541K^ induces the expression of pathogenicity traits in strain 101.** **A.** Representative images of filamentation *C. albicans* strains 101 wild type, 101_*CYR1*^E1541K^, and 101_*CYR1*^E1541K/E1541K^ and SC5314 wild type put in contact with a monolayer of TR146 keratinocytes for 3.5 – 4 hours at 37°C, 5% CO_2_. **B.** *HWP1*, *ALS3*, and *NRG1* expression levels by the fungal strains after exposure to TR146 keratinocytes for 24 hours at 37°C, 5% CO_2_. Each symbol represents one sample. Data are from one representative out of two independent experiments with three samples per strain each. The mean ± SD is indicated. **C.** *HWP1* and *NRG1* expression levels in the tongue tissue of C57BL/6 mice on day 1 after colonization with 101 wild type or 101_*CYR1*^E1541K^. Data are pooled from two independent experiments. The mean ± SEM is indicated. Statistical significance was determined using one-way ANOVA (B) or unpaired Student’s *t* test (C). *p<0.05, **p<0.01, ***p<0.001, ****p<0.0001.

**
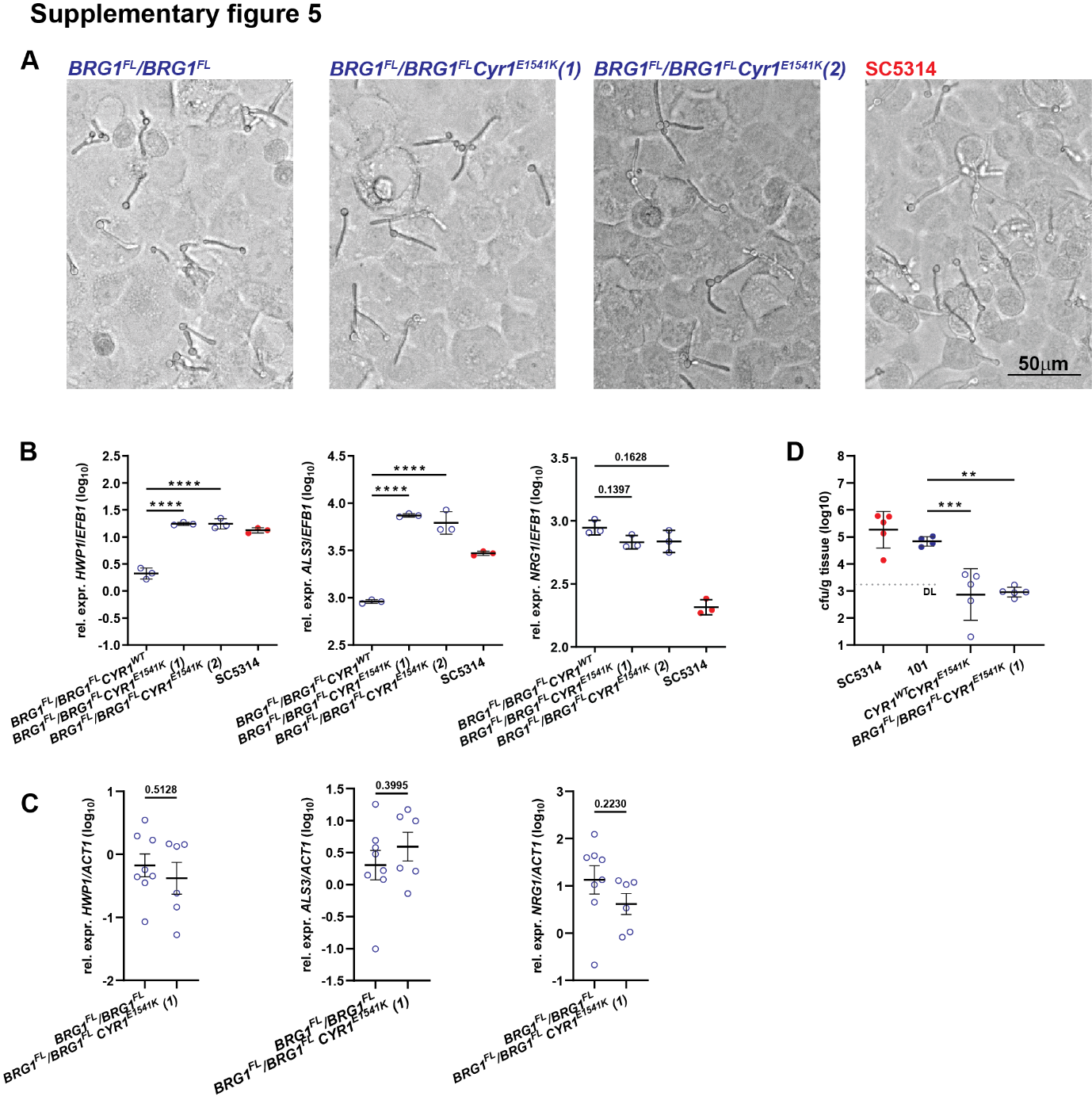
**

**Figure S5 (related to Figure 4).** ***CYR1*^E1541K^-induced pathogenicity traits are independent of the *BRG1* locus.** **A.** Representative images of filamentation *C. albicans* strains 101_*BRG1*^FL/^*BRG1*^FL^, 101_*BRG1*^FL/^*BRG1*^FL^ *CYR1*^E1541K^ (clone 1 and clone 2), and SC5314 wild type upon contact with a monolayer of TR146 keratinocytes for 3.5 – 4 hours at 37°C, 5% CO_2_. **B.** *HWP1*, *ALS3* and *NRG1* expression levels by the fungal strains after exposure to TR146 keratinocytes for 24 hours at 37°C, 5% CO_2_. Each symbol represents one sample. Data are from one representative out of two independent experiments with three samples per strain each. The mean ± SD is indicated. **C.** *HWP1*, *ALS3* and *NRG1* expression levels in the tongue tissue of C57BL/6 mice on day 7 after colonization with 101_*BRG1*^FL/^*BRG1*^FL^ or 101_*BRG1*^FL/^*BRG1*^FL^ *CYR1*^E1541K^ (clone 1). Each symbol represents one mouse. Data are pooled from two independent experiments. The mean ± SEM is indicated. **D.** Kidney CFU in mice intravenously infected with *C. albicans* strains SC5314, 101, 101_*CYR1*^E1541K^ or 101_*BRG1*^FL/^*BRG1*^FL^ *CYR1*^E1541K^ (clone 1) for 3 days. Each symbol represents one mouse. DL, detection limit. Statistical significance was determined using one-way ANOVA (B) or unpaired Student’s *t* test (C). *p<0.05, **p<0.01, ***p<0.001, ****p<0.0001.

**Supplementary Table S1. Genome comparison of strain 101_BRG1^FL^/BRG1^FL^(*) to strain 101_BRG1^FL^/BRG1^FL^**. Yellow-coloured SNPs indicate non-synonymous mutations; the red-coloured position indicates the mutation in *CYR1*. All other SNPS are intergenic, synonymous or not unique in the set of 298 clinical isolates.

**Supplementary Table S2. Spontaneous mutations in strains 101 and 101_BRG1^FL^/BRG1^FL^**. Green-coloured position indicates a mutation in *RAS1*; red-coloured positions indicate non-synonymous SNPs in the same ORF; yellow-coloured positions indicate SNPs in ORFs occurring in all datasets; non-coloured positions indicate single non-synonymous SNPs in ORFs.
